# Surface protein profiling of milk and serum extracellular vesicles unveils body fluid-specific signatures

**DOI:** 10.1038/s41598-023-35799-w

**Published:** 2023-05-30

**Authors:** Alberta Giovanazzi, Martijn J. C. van Herwijnen, Marije Kleinjan, Gerbrich N. van der Meulen, Marca H. M. Wauben

**Affiliations:** 1 Department of Biomolecular Health Sciences, Faculty of Veterinary Medicine, Utrecht University, Utrecht, The Netherlands; 2 TRAIN-EV Marie Skłodowska-Curie Action-ITN, Utrecht, The Netherlands; 3 Department of Paediatrics, Paediatric Allergy Centre, Martini Hospital, Groningen, The Netherlands

**Keywords:** Cell biology, Biomarkers

## Abstract

Cell-derived extracellular vesicles (EVs) are currently in the limelight as potential disease biomarkers. The promise of EV-based liquid biopsy resides in the identification of specific disease-associated EV signatures. Knowing the reference EV profile of a body fluid can facilitate the identification of such disease-associated EV-biomarkers. With this aim, we purified EVs from paired human milk and serum samples and used the MACSPlex bead-based flow-cytometry assay to capture EVs on bead-bound antibodies specific for a certain surface protein, followed by EV detection by the tetraspanins CD9, CD63, and CD81. Using this approach we identified body fluid-specific EV signatures, e.g. breast epithelial cell signatures in milk EVs and platelet signatures in serum EVs, as well as body fluid-specific markers associated to immune cells and stem cells. Interestingly, comparison of pan-tetraspanin detection (simultaneous CD9, CD63 and CD81 detection) and single tetraspanin detection (detection by CD9, CD63 or CD81) also unveiled body fluid-specific tetraspanin distributions on EVs. Moreover, certain EV surface proteins were associated with a specific tetraspanin distribution, which could be indicative of the biogenesis route of this EV subset. Altogether, the identified body fluid-specific EV profiles can contribute to study EV profile deviations in these fluids during disease processes.

## Introduction

Extracellular vesicles (EVs) are submicron lipid bilayer-delimited particles naturally released by cells that act as mediators of inter-cellular communication by targeting biologically active molecules to adjacent and distant cells^1^. Cells in body tissues communicate by releasing EVs into proximal body fluids, such as breast milk and blood^2^. Circulating EVs can originate from cells present in the body fluids, cells lining the cavities of extruded fluids or from tissue-resident cells^2^, and for this reason they can carry body fluid-specific and tissue-specific signatures. Additionally, the molecular make-up of EVs can be affected by the status of their originating cells and, as such, EVs can be enriched or depleted for specific surface proteins, resulting in specific protein biomarker profiles associated with (patho)physiological conditions^3,4^. Nowadays, EV-based biomarker discovery attracts a lot of attention for monitoring disease and health status. The promise of EV-based biomarkers resides in the unique combination of different EV molecules, resulting in a “combined” biomarker that outperforms single component-based biomarkers. To identify EV-based biomarkers of disease or disturbed homeostasis, knowing the “normal” molecular reference profile of EVs in different body fluids is of utmost importance. However, the discovery of such body fluid-specific EV profiles is complicated due to the colloidal properties of body fluids, containing non-EV particles with overlapping characteristics of EVs^5,6^. For example, lipoproteins in blood and casein micelles in milk, which co-isolate to various degrees with EVs^7,8^, can act as confounders in (semi)-quantitative EV analyses. Hence for comparative analyses of EVs present in different body fluids, a tailored protocol for EV isolation might overcome these problems.

In immunoassays developed for EV phenotyping, the tetraspanins CD9, CD63 and CD81 are commonly used as *bona fide* EV-associated markers for “total” EV detection. These tetraspanins have primary functions in EV formation, cargo selection/sorting and EV release and uptake^9^. Via their extracellular domains, tetraspanins associate with other tetraspanins and surface proteins thereby forming “tetraspanin webs” resulting in membrane domains with a variety of surface protein profiles^10^. Importantly, in recent years, it has been reported that the distribution of tetraspanins is more heterogeneous than assumed across single EVs^11–14^. Moreover, specific combinations of CD9, CD63 and/or CD81 on EVs can give insights on their specific biogenesis route^11,12^. Consequently, with the purpose of analysing EV surface protein profiles, the common approach to detect EVs via the simultaneous use of EV-associated CD9, CD63 and CD81 can mask important biological information and can even hamper the identification of unique EV protein profiles.

In the present study, we isolated EVs from paired human milk and serum samples by using a similar method based on validated protocols for minimal non-EV particle contaminations^15–18^. Comparative analyses of surface protein profiles of milk and serum EVs were performed by multiplexed flow cytometric bead assay (MACSPlex) based on the capture of EVs by antibodies for specific surface proteins, and pan-tetraspanin or individual tetraspanin detection of bead-bound EVs. Using this approach we were able to identify milk and serum-specific EV-associated protein profiles and gaininsights on the tetraspanin composition in relation to these proteins and the possible biogenesis routes of specific EV subsets.

## Results

### Concentration and size determination of EVs isolated from paired human milk and serum samples

**Figure 1:**
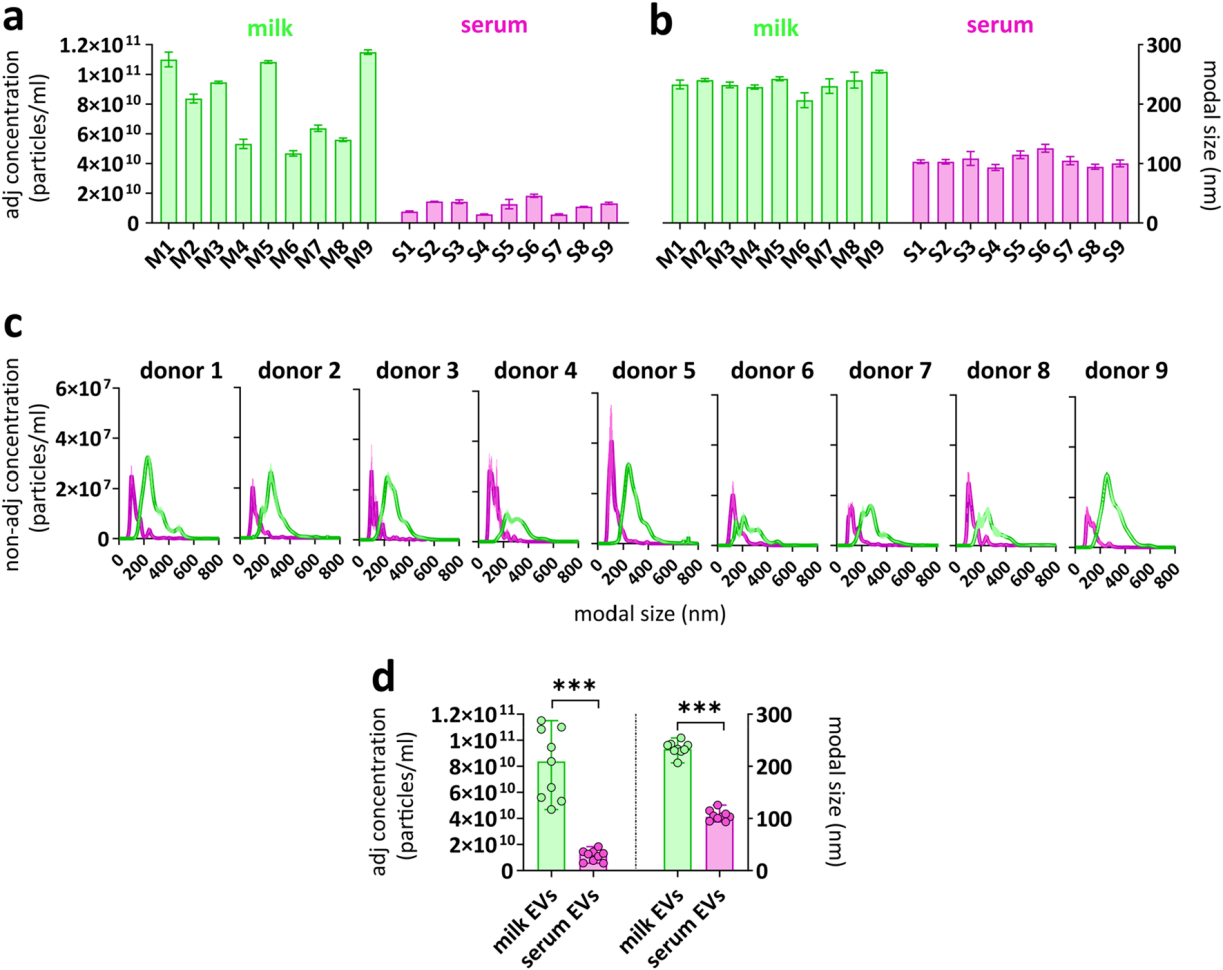
Concentration and size distribution of paired milk EV and serum EV samples. NTA was used to determine (**a**) the adjusted nanoparticle concentration (particles/ml) and (**b**) the modal size (diameter) of n = 9 paired milk EV and serum EV samples. Donors 2, 4, 6, 8 and 9 were non-allergic donors and donors 1, 3, 5 and 7 were allergic donors. Bars represent mean ± SD from three captures of 60 s per sample. (**c**) NTA histograms show the non-adjusted concentration and the size distribution of paired milk EVs (green) and serum EVs (pink). (**d**) The median adjusted nanoparticle concentration and modal size of n = 9 paired milk EV samples and serum EV samples. Significance was tested by 2-tailed non-parametric Mann–Whitney T test, p < 0.001 (***).

To define body fluid-specific EV profiles, paired human milk and serum samples donated on the same day by non-allergic or allergic mothers were used. For comparative analysis of milk and serum-derived EVs, EVs from both body fluids were isolated using our previously optimized milk EV isolation protocol, starting with differential centrifugation until 10,000×*g* to avoid the formation of EV-containing aggregates^15,16,19,20^. Subsequently, non-EV colloidal structures were depleted by density gradient separation followed by size exclusion chromatography as described before^15,16^ and isolated EV-containing fractions were checked for the presence of CD9, CD63 and CD81 by western blotting (Supplementary Fig. S1). Since equal amounts of EVs are crucial for reliable comparisons of multiplex bead-based flow cytometric analysis of EV surface proteins^21^, next we determined EV concentration and size by Nanoparticle Tracking Analysis (NTA). For reliable NTA analyses^22^, only measurements with at least 1000 valid tracks per EV sample and 10–100 particles in frame were processed (Supplementary Fig. S2). The concentration of milk EVs isolated from n = 9 individual donors ranged between 5 × 10^10^ and 1.2 × 10^11^ particles/ml (average ± standard deviation = 8 × 10^10^ ± 2.8 × 10^10^), while the concentration of serum EVs ranged from 5 × 10^9^ to 1.5 × 10^10^ particles/ml (average ± standard deviation = 1 × 10^10^ ± 4.3 × 10^10^) (Fig. 1a). The modal size of milk EVs and serum EVs was in the range of 230 ± 13 nm and 105 ± 10 nm, respectively (Fig. 1b). Size distribution histogram overlays of paired milk and serum EV samples show this size difference for each individual donor (Fig. 1c). No significant differences in particle concentrations and size distributions were observed between EVs derived from non-allergic and allergic donors. In contrast, milk EV samples had significantly higher particle concentration and bigger size than the paired serum EV samples (Fig. 1d).

### Optimization of the MACSPlex analysis for comparative surface protein profiling of purified milk and serum EVs

To study milk and serum EV surface proteins, the multiplex bead-based flow cytometry MACSPlex was used. The principle of this assay relies on the use of hard-dyed bead populations, each coupled to different antibodies that recognize 37 potential EV surface antigens (and 2 internal isotype controls)^21,23^. Beads are distinguished by their specific fluorescence characteristics (FITC VS PE) and binding of EVs is detected by APC-conjugated antibodies targeting the gold standard EV markers CD9, CD63 and CD81 (Pan-tetraspanin detection) (APC VS FITC) (Supplementary Fig. S3).

**Figure 2:**
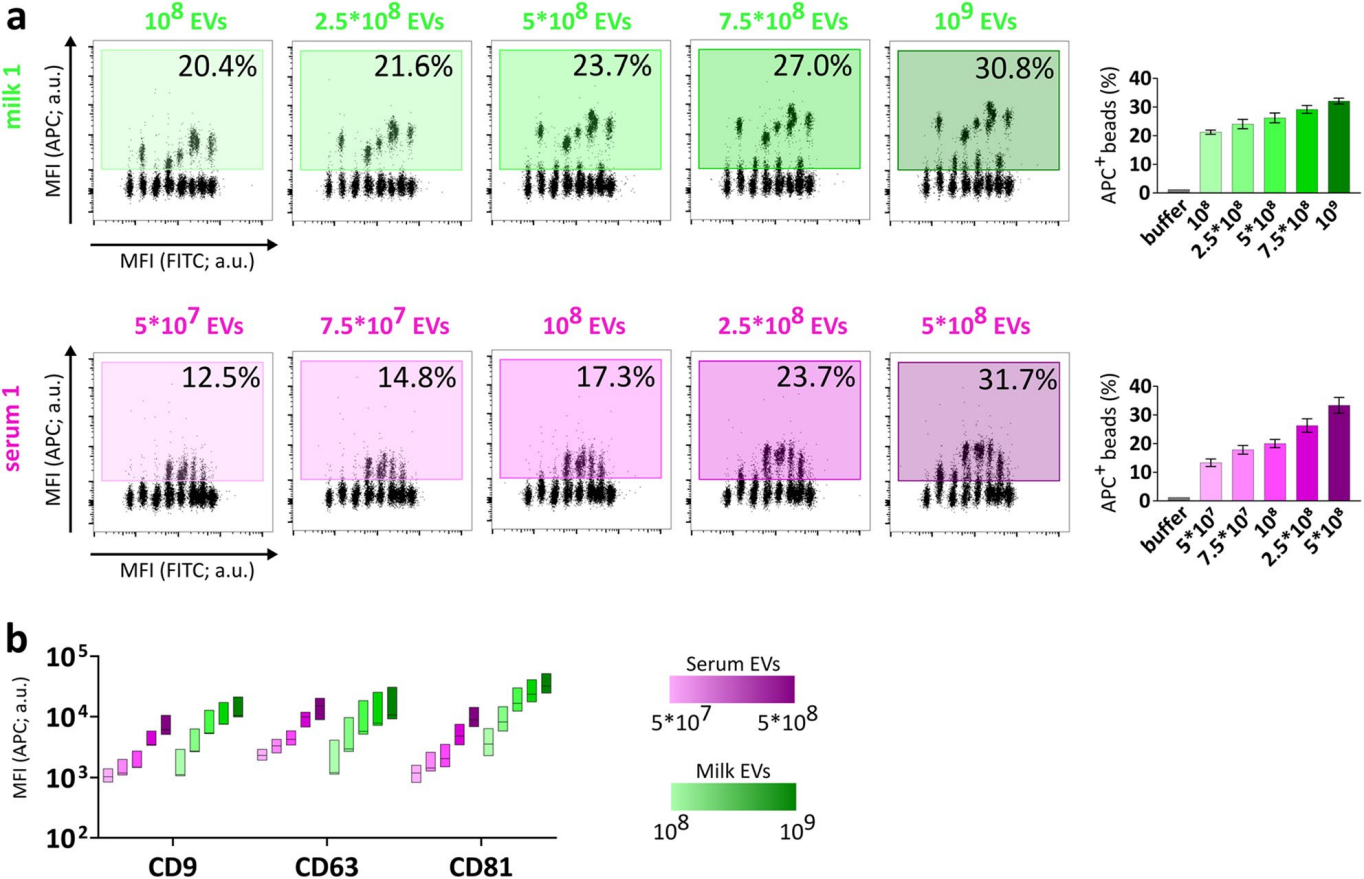
Titration of APC detection signals is proportional to input EV numbers. (**a**) Flow cytometry dot plots of five input amounts of milk EVs (green) and serum EVs (pink) from donor 1 depicting the percentage of APC^+^ beads (relative to single beads) (For donors 2 and 3, see Supplementary Fig. S3). The bar graphs show the percentage of APC^+^ beads as mean ± SD of three donors (donor 1, donor 2, donor 3). (**b**) The floating bar graph depicts the median + min and max values (Donors 1–3) of MFI APC relative to CD9, CD63, CD81 capture beads at different EV doses. a.u. = arbitrary unit.

To perform a robust comparative analysis, we first defined the optimal input dose of EVs by testing 5 different amounts, ranging from 10^8^ to 10^9^ EVs purified from milk and 5 × 10^7^–5 × 10^8^ EVs purified from serum (Supplementary Table S2). As expected, the percentage of APC^+^ events proportionally increased with increasing input numbers of EVs (Fig. 2a, Supplementary Fig. S3). When comparing the percentage of total APC^+^ events between the same amounts of serum and milk EVs, no significant differences were observed, corroborating the defined input dose of EVs based on the NTA measurements (Supplementary Fig. S3). Moreover, the median APC signal associated to the tetraspanin capture beads increased by approximately 10X from the lowest to the highest EV dose tested and thus showed a proportional titration for both serum and milk samples, while the signal intensities for the internal isotype control bead populations were marginally affected with increasing EV amounts (Fig. 2b and Supplementary Fig. S3). Since 5 × 10^8^ was the highest dose of serum EVs that could be obtained without further sample concentration and which resulted in robust and clear APC detection signals, we selected 5 × 10^8^ input EVs for further comparative analyses of milk and serum EVs. Overall, these results showed that the MACSPlex assay is suitable for comparative analysis of EVs purified from human milk and serum.

### Pan-tetraspanin EV detection unveils body fluid-specific protein signatures

**Figure 3:**
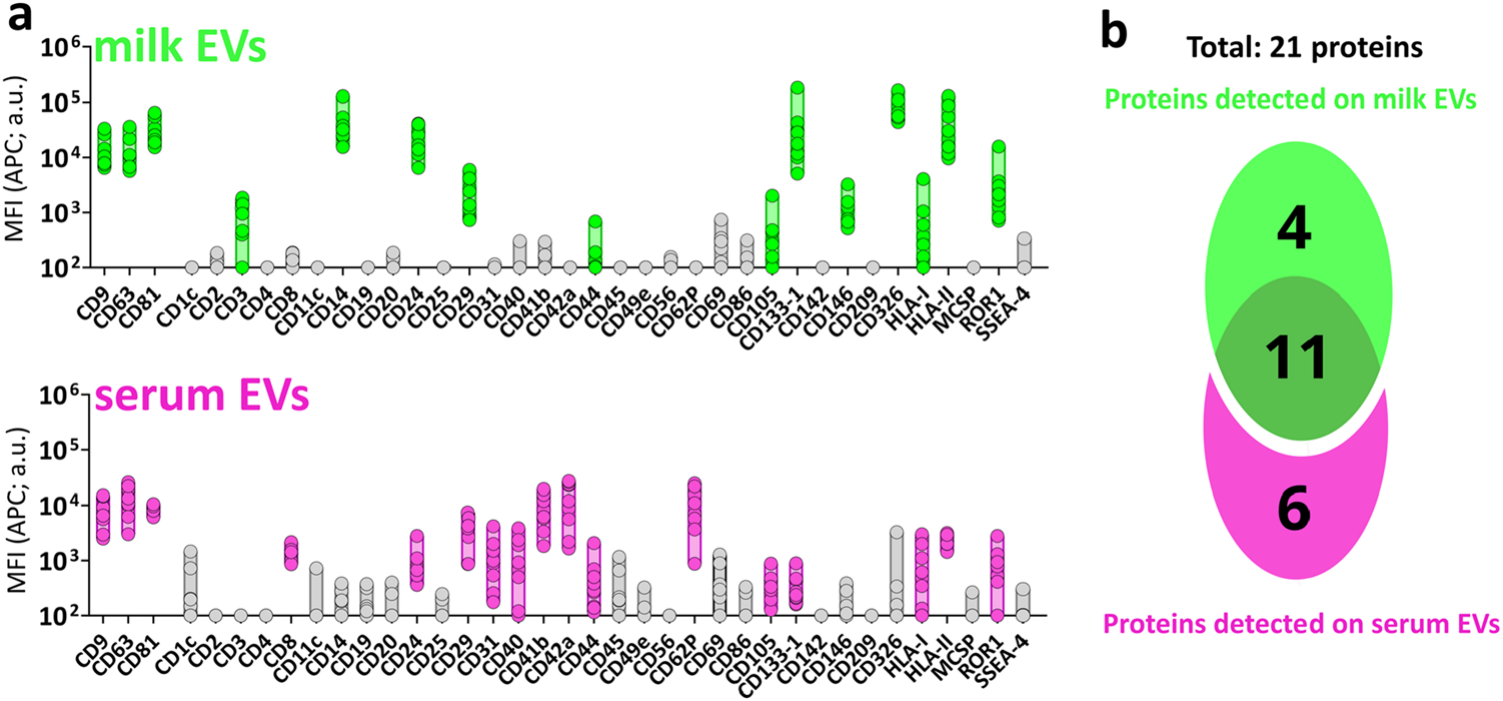
Protein distribution on milk EVs and serum EVs with pan-tetraspanin detection. (**a**) MFI APC signal of pan-tetraspanin detection associated to the 37 MACSPlex proteins in n = 9 paired milk EV (green) and serum EV (pink) samples (5 × 10^8^ EVs). In grey proteins with associated MFI APC signals not significantly higher than the MFI APC of the corresponding internal isotype control (non-detected). For statistical analysis see Supplementary Table S4 in which significance was determined by non-parametric Kruskal–Wallis Test with post-hoc Dunn’s multiple comparison. (**b**) Venn Diagram depicting unique and common proteins of milk EVs and serum EVs. a.u. = arbitrary unit.

After the optimization of the MACSPlex assay for the comparative analyses, we performed surface protein profiling of nine paired milk and serum EV samples by pan-tetraspanin detection (i.e., EV detection by using simultaneously antibodies targeting CD9, CD63 and CD81) (For raw data see: Supplementary Table S3). Proteins with Median Fluorescent Intensity of the APC signal (MFI APC) lower than the respective isotype controls were considered non-detected (Fig. 3a and Supplementary Table S4). Based on this criterium, 15 and 17 proteins were detected on EVs from milk and serum, respectively (Fig. 3b), with a total number of 21 detected proteins. No significant differences in MFI APC signal associated to the 21 detected proteins were observed between EVs from non-allergic donors (n = 5) and allergic donors (n = 4) (Supplementary Fig. S4).

**Figure 4:**
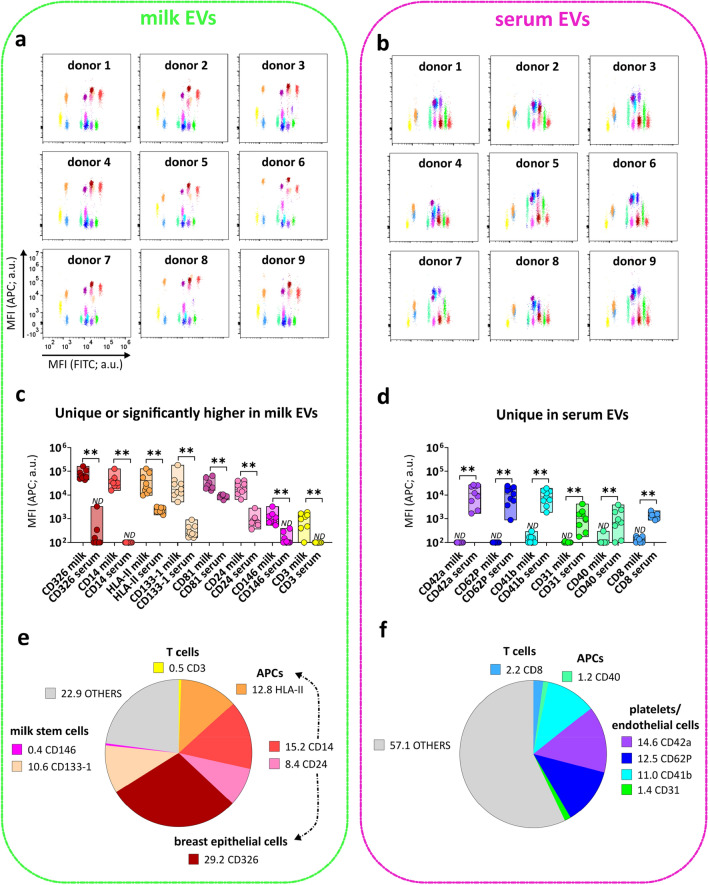
Surface proteins uniquely or differentially expressed on milk EVs and serum EVs. Flow cytometry dotplots from milk EVs (**a**) and serum EVs (**b**) representing all the 14 proteins enriched either in milk EVs (8 proteins) (**c**) or in serum EVs (6 proteins) (**d**). Bars indicate minimum and maximum value with median of MFI APC pan-tetraspanin values from n = 9 EV samples per body fluid. Statistical significance was determined by multiple non-parametric matched pairs Wilcoxon test with Benjamini and Hochberg FDR method, p < 0.01 (**). Percentage expression of the total MFI APC pan-tetraspanin signal (MFI APC values of each capture bead population was converted into a percentage of the total signal) for proteins enriched in milk EVs (**e**) and in serum EVs (**f**). Proteins are clustered based on known cellular markers. “Others” (indicated in grey) includes protein signals lower than the other body fluid (e.g. CD133, HLA-II, CD81, CD24 for serum EVs), non-significantly different proteins (Supplementary Fig. S5a) and proteins below the isotype threshold (Fig. 3). ND is non-detectable as defined by the isotype threshold (Fig. 3). a.u. = arbitrary unit.

Eleven out of the 21 detected proteins were in common between milk and serum EVs and no significant differences were observed in the MFI APC pan-tetraspanin signals of 7 proteins (CD9, CD63, HLA-I, CD29, CD44, CD105, ROR1) (Supplementary Fig. S5). The flow cytometry dotplots representing the MFI APC of the pan-tetraspanin detection of the remaining 14 detected proteins show different profiles in milk EVs and serum EVs (Fig. 4a,b). Specifically, the pan-tetraspanin detection levels of CD326, CD14, HLA-II, CD133-1, CD81, CD24, CD146 and CD3 were significantly higher in milk EVs, and 4 proteins were exclusively detected on milk EVs (CD326, CD14, CD146 and CD3) (Fig. 4c). The pan-tetraspanin signals for CD42a, CD62P, CD41b, CD31, CD40, CD8 were only detected in serum EVs and undetectable in milk EVs (Fig. 4d). Taken together, these results show that besides common surface proteins, milk EVs and serum EVs also express body fluid-specific surface proteins.

Next, we attempted to identify the potential cells of origin of the body fluid-specific/enriched EV proteins present in milk and serum (Fig. 4e,f). In milk EVs, 11% of the total MFI APC signal from the pan-tetraspanin detection of the assay was derived from CD133-1 (10.6%) and CD146 (0.4%), which could be indicative of a breast milk stem cell origin^24–27^. The CD326 signal corresponding to 29.2% of the total APC signal might point towards EVs originating for luminal breast epithelial cells (lactocytes or milk secretory cells)^8,28^, while the MHC-II and CD24 signals, respectively 12.8% and 8.4% of the total APC signal, hint towards the presence of antigen-presenting cell-derived EVs in milk. However, caution should be taken when interpreting these results since most surface proteins are expressed on several different cell types and similarities exist between certain leukocytic and breast epithelial cell populations^29,30^. For example, CD14, despite being first identified as marker of mature monocytes, can be associated to mammary epithelial cells^31^. CD24, often associated to B cells, in the context of breast milk can also mark luminal epithelial cell-derived EVs^32^. Additionally, the glycoprotein CD44^25,33^ and Integrinβ1/CD29^25^ are reported to be widely expressed on basal myoepithelial breast cells (from the ducts and alveoli of mammary gland) and CD44 has also been described as a marker of stem cells in milk^26^. However, because of the broad expression of CD44 on many different cell types, tracing the cell of origin of CD44^+^ EVs without additional cell type-specific markers is not possible. In contrast, the low but unique expression of the T cell marker CD3 on milk EVs (0.5% of total APC signal), strongly suggests the presence of T cell-derived EVs in milk. Overall, these data show that 77.1% of the total pan-tetraspanin APC signal from milk EVs can be attributed to surface proteins uniquely or significantly higher expressed on EVs from milk compared to serum (Fig. 4e).

In serum EVs, 39.5% of the total pan-tetraspanin APC signal accounted for platelets-associated markers, i.e. CD41b (11%) and CD42a (14.6%), and ongoing platelet activation and interaction with endothelium (P-selectin CD62P (12.5%) and platelet endothelium adhesion molecule PECAM-1/CD31 (1.4%)^34^. These results suggest that EVs derived from activated platelets, as expected, are highly abundant in serum (Fig. 4f). Besides platelet signatures in serum EVs, serum-specific proteins corresponding to antigen presenting cells (i.e. CD40 1.2% of total MFI APC pan-tetraspanin signal) and T cells (i.e. CD8 2.2% of total MFI APC pan-tetraspanin signal) were detected. Overall, comparative milk and serum EV analysis shows that the serum-specific proteins make up 42.9% of the total MFI APC pan-tetraspanin signal of the array.

### Tetraspanins are heterogeneously distributed on EVs from milk and serum

**Figure 5:**
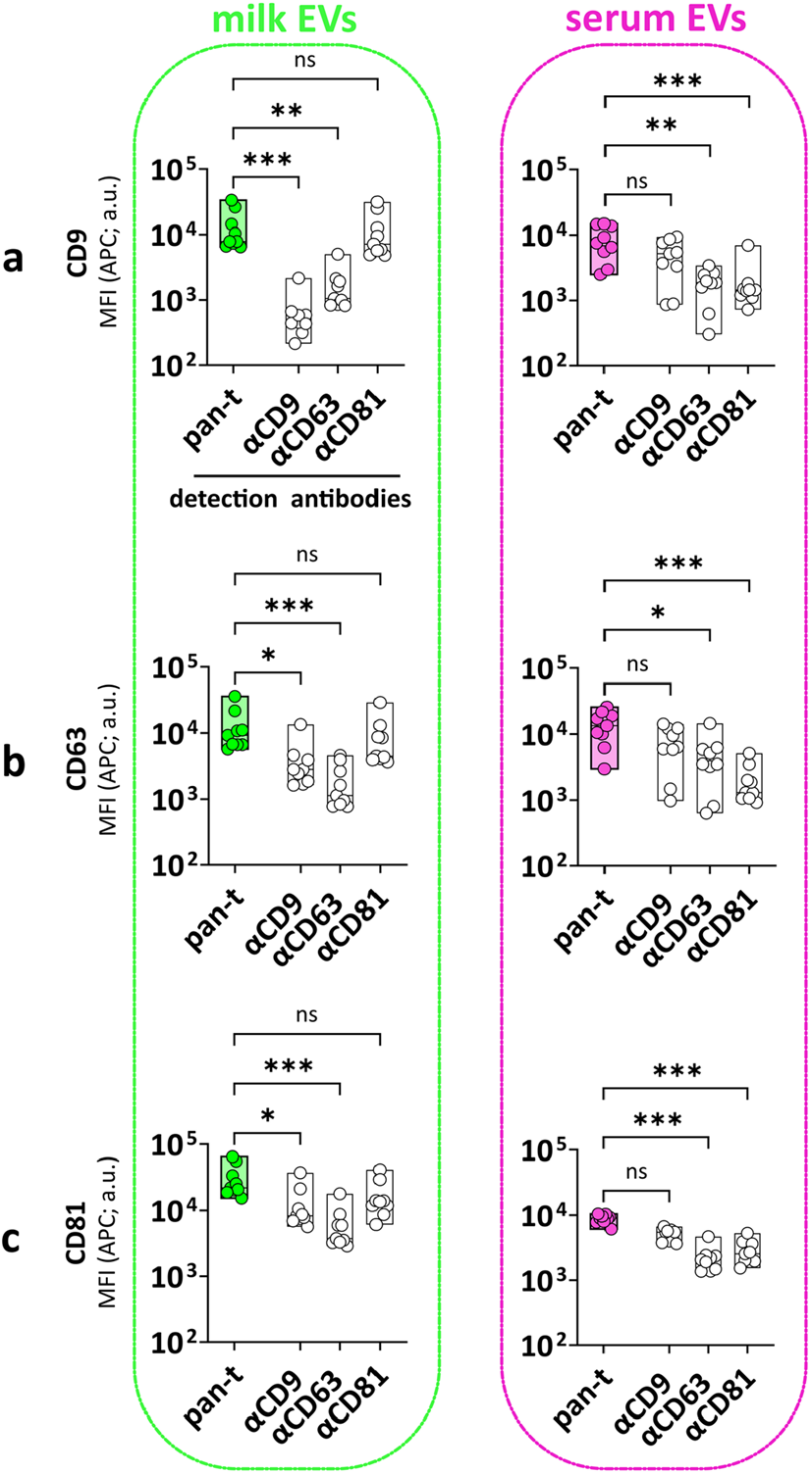
Heterogeneous distribution of tetraspanins on EVs from milk and serum. MFI APC of CD9 (**a**), CD63 (**b**) and CD81 (**c**) capture bead populations (x-axis) detected with standard pan-tetraspanin antibody (pan-t: αCD9, αCD63 and αCD81) or single tetraspanin antibody (αCD9, αCD63 or αCD81) (y-axis). Bar graphs delimited by minimum and maximum value with median of MFI APC values from n = 9 EV samples per body fluid. Non-parametric Kruskal–Wallis Test with post-hoc Dunn’s multiple comparison was performed to compare pan-tetraspanin with each single tetraspanin detection, p ≥ 0.05 (ns), p < 0.05 (*), p < 0.01 (**), p < 0.001 (***). a.u. = arbitrary unit.

In the pan-tetraspanin detection, the fluorescence signal from all three CD9, CD63 and CD81 tetraspanins was used to detect EVs. However, tetraspanins are unequally distributed on EV subsets^11,12^, as also demonstrated in the pan-tetraspanin detection for the CD81 capture bead, showing significantly higher signals in milk EVs compared to serum EVs, both in the group analysis (Fig. 4c and Supplementary Fig. S6), as well as in the paired analysis of individual donors (Supplementary Fig. S6). To explore EV subset differences between milk and serum in greater detail, next we compared pan-tetraspanin detection (αCD9, αCD63 and αCD81) with single tetraspanin detection (αCD9, αCD63 or αCD81). While the CD9 capture bead signal based on pan-tetraspanin detection was not significantly different between serum EVs and milk EVs (Supplementary Fig. S6), the single tetraspanin detection showed clear differences between EVs from serum and milk. Albeit all tetraspanins could detect EVs captured on CD9-beads, the main contributor to the CD9 bead capture signal in the pan-tetraspanin detection differed between milk and serum; i.e. CD81 in milk (pan-t VS αCD81, p ≥ 0.05, ns) and CD9 in serum (pan-t VS αCD9, p ≥ 0.05, ns) (Fig. 5a and Supplementary Fig. S7). Similar patterns were also observed for the CD63 and CD81 capture bead signals, for which respectively no significant differences and significant differences between serum and milk EVs were observed in the pan-tetraspanin detection (Supplementary Fig. S6). For milk EVs CD81 was the main contributor to the pan-tetraspanin CD63 and CD81 capture bead signals, while CD9 was in serum EVs (Fig. 5b,c and Supplementary Fig. S7). In conclusion, single tetraspanin detection revealed body fluid-specific signatures on EVs that were masked in pan-tetraspanin detection.

### Single tetraspanin detection of milk EVs and serum EVs unveils different tetraspanin profiles depending on the capture antibody

**Figure 6:**
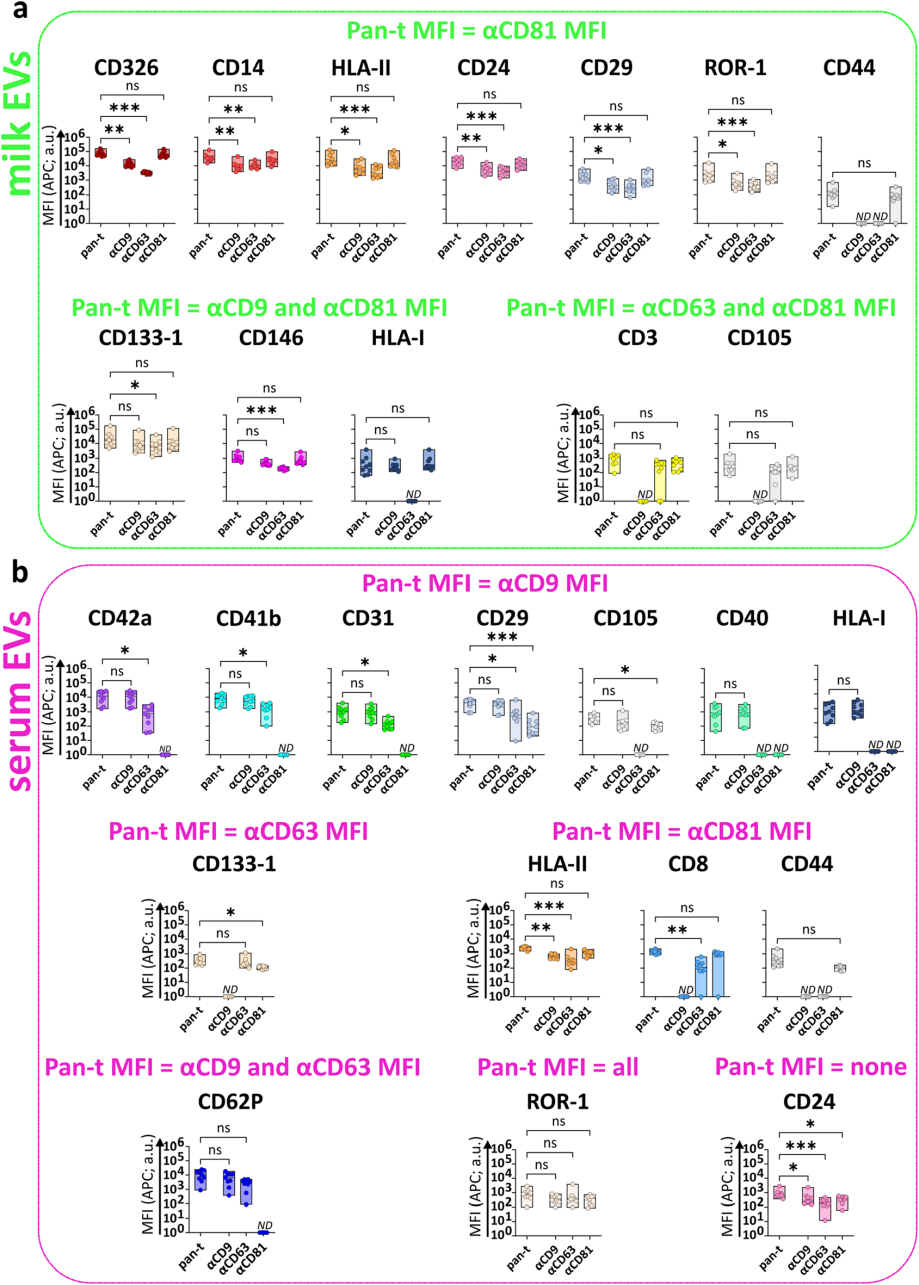
Comparison of pan-tetraspanin and single tetraspanin detection of milk EVs and serum EVs bound to different protein capture bead populations. MFI APC signals of capture bead populations (identified in Fig. 3) on milk EVs (**a**) and serum EVs (**b**) from pan-tetraspanin detection (pan-t: αCD9, αCD63 and αCD81) in comparison to each individual tetraspanin detection (αCD9, αCD63 or αCD81). Bar graphs delimited by minimum and maximum value with median of MFI APC values from n = 9 EV samples per body fluid. Non-parametric Kruskal–Wallis Test with post-hoc Dunn’s multiple comparison was performed to compare pan-tetraspanin with each single tetraspanin detection, p ≥ 0.05 (ns), p < 0.05 (*), p < 0.01 (**), p < 0.001 (***). a.u. = arbitrary unit.

To unveil possible body fluid-specific tetraspanin distributions on EVs expressing the surface proteins identified in Fig. 3, we compared pan-tetraspanin with single tetraspanin detection signals (Fig. 6a,b and Supplementary Fig. S8). For milk EVs (Fig. 6a), we identified 7 proteins/capture bead populations (i.e. CD326, CD14, HLA-II, CD24, CD29, ROR-1 and CD44) for which the MFI APC pan-tetraspanin detection signal did not significantly differ from the single CD81 detection signal, suggesting that the signal from CD81 detection was the main contributor to the pan-tetraspanin signal of EVs bound to these capture bead populations. For 6 out of these 7 bead populations (i.e. CD326, CD14, HLA-II, CD24, CD29 and ROR-1), single CD9 and single CD63 detection signals were observed but were significantly lower than the pan-tetraspanin detection. In contrast, the CD9 and CD63 single detection levels of EVs bound to the CD44 capture bead were below the isotype threshold, indicating that these EVs only expressed CD81. For 3 other milk EV markers (i.e. CD133-1, CD146 and HLA-I) single CD9 and single CD81 detection contributed equally to the pan-tetraspanin signal. In contrast to CD133-1 and CD146, milk EVs captured by HLA-I did not express CD63 (below the isotype threshold). Finally, the MFI APC pan-tetraspanin signal of milk EVs captured by CD3 and CD105 was comparable to single CD63 and CD81 detection, while these EVs did not express CD9 (below the isotype threshold).

For serum EVs (Fig. 6b), the pan-tetraspanin detection signal of 7 capture bead populations (i.e. CD42a, CD41b, CD31, CD29, CD105, CD40 and HLA-I) did not significantly differ from single CD9 detection signal. For the CD40 and HLA-I capture bead populations both CD63 and CD81 single tetraspanin detections were below the isotype control, indicating that CD40^+^ and HLA-I^+^ serum EVs only expressed CD9. For CD42a, CD41b, CD31 and CD62P capture bead populations, besides CD9, also CD63 single detection signals were measured, while CD81 was not detected. Conversely, serum EVs bound to CD105 capture bead, besides CD9, also expressed CD81 but were negative for single CD63 detection. EVs expressing CD29, ROR1, CD24 and HLA-II were detected with all single tetraspanin antibodies. Remarkably, serum EVs expressing CD8, CD44 and CD133-1 lack CD9 and the main contributor or even sole contributor to the pan-tetraspanin signal was respectively, CD81 for CD8 or CD44-expressing EVs and CD63 for CD133-1-expressing EVs.

Comparison of the tetraspanin signals on EVs positive for markers detected in both milk and serum EVs (i.e. HLA-I, HLA-II, CD24, CD29, CD44, CD105, CD133-1 and ROR1) unveiled similar tetraspanin signals for EVs captured by HLA-II, CD24, CD29 or ROR1, expressing all 3 tetraspanins. Also on CD44^+^ EVs the tetraspanin expression is similar between milk and serum, but these EVs lack CD9 and CD63. For both milk and serum HLA-I EVs, CD63 was not detected and CD9 contributed largely to the pan-tetraspanin signal. However, in contrast to HLA-I^+^ milk EVs, which also expressed CD81, CD81 was not detected on HLA-I^+^ serum EVs. For CD133-1-expressing EVs, in contrast to milk EVs, serum EVs were negative for CD9. Conversely, CD105-expressing milk EVs were negative for CD9, while CD105 serum EVs were negative for CD63 (Fig. 6a,b).

In conclusion, single tetraspanin detection unveiled EV subset-specific tetraspanin profiles and allowed the identification of similar and different tetraspanin distribution on milk and serum-derived EVs.

**Figure 7:**
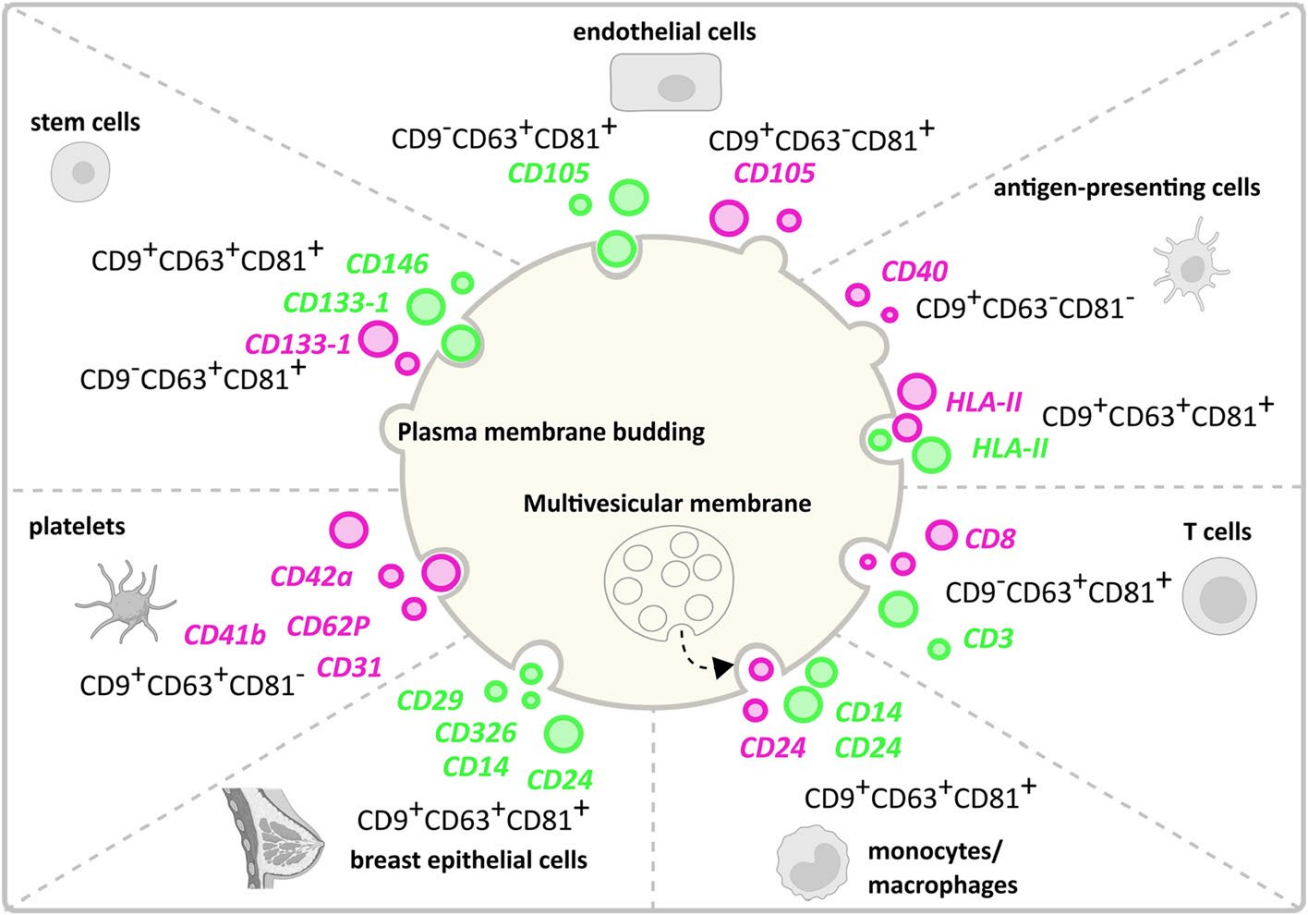
Graphical representation of a hypothetical model showing the potential cells of origin and biogenesis route of milk EVs and serum EVs. This model connects potential cells of origin data described in Fig. 4 and the tetraspanin distributions described in Fig. 6. Milk EV (green) and serum EV (pink) cell markers are depicted in combination to their association with CD9, CD63 and CD81, from which we propose their biogenesis route based on Kowal et al*.*^11^ and Mathieu et al*.*^12^ study. Created with BioRender.com and Inkscape 1.2.1.

Finally, based on our extrapolation of possible cells of origin of EVs as described in Fig. 4 and the tetraspanin distributions described in Fig. 6, we present a hypothetical model representing the potential cells of origin of EVs expressing a certain marker connected to proposed biogenesis routes for that EV subset (Fig. 7). Based on EV proteomics analyses and intracellular trafficking, Kowal et al*.*^11^ and Mathieu et al*.*^12^ recently proposed the use of combinations of CD9, CD63 and/or CD81 for the definition of EV subsets on the basis of their intracellular origin. More specifically, CD9^+^CD63^+^CD81^+^ EVs and CD63^+^CD9^low^ EVs carrying endosomal proteins qualify as exosome (endosomal origin); CD9^+^CD63^−^CD81^−^ and CD9^+^CD81^+^CD63^low^ small EVs mainly bud from the plasma membrane. The subpopulations of CD9 single positive and CD81 single positive small EVs probably form at the plasma membrane and early endocytic locations. Platelet-derived EVs characterized by a CD9^+^CD63^+^CD81^−^ profile are more difficult to classify following the criteria by Kowal et al*.*^11^ and Mathieu et al*.*^12^. However, based on previous observations indicating that platelet-derived EVs in the size range of 30-100 nm, expressing CD9, CD63, TSG101, ALIX, CD31, CD41b, CD42a, CD62P and PF4 originate from multi-vesicular bodies^35,36^, in our model we classified platelet-derived serum EVs as endosomal-derived.

## Materials and methods

### Milk and serum donors

Human milk and serum were collected upon written consent from lactating women who had given birth to a full-term newborn via vaginal delivery. These women were enrolled in the Comparison of Human Milk Extracellular Vesicles in Allergic and Non-allergic Mothers (ACCESS) study (NL 47426.099.14; RTPO 914), approved by the Martini Hospital Medical Ethics Committee (Groningen, the Netherlands) in accordance with the local ethical guidelines and the Declaration of Helsinki for medical research involving human subjects. Informed consent to be included in the ACCESS study was obtained from all donors. Mothers were classified as “allergic” when total serum IgE ≥ 50 kU/L and/or specific IgE was detected for grass pollen, tree pollen, house dust mite, cat dander, or dog dander by positive (≥ 0.35 kU/L) Phadiatop assay (Thermo Scientific, Uppsala, Sweden). In clinical practice, 0.35 kU/L has commonly been used as a cut off (< 0.35 kU/L is considered < limit of detection -LOD-). For this paper, paired milk and serum from n = 9 women (n = 4 allergic; n = 5 non-allergic) were selected based on sample availability. Information on donors is reported in Supplementary Table S1.

### Human milk and serum collection

Paired milk and serum samples were collected at the same day during a hospital check-up visit. Milk was collected in sterile Medela's BPA-free human milk collection containers, as previously described^15^. Shortly, milk was prevented from cooling down and was processed within 20 min after collection by 2 rounds of centrifugation at 3000×*g* for 10 min at 23 °C (Beckman Coulter Allegra X-12R, Fullerton, CA, USA) to deplete cells and fat. Next, cell and fat-free milk supernatants were stored at − 80 °C. Peripheral blood (6 ml) was collected in serum-separating tubes. Blood was allowed to clot by leaving it undisturbed for 30 min at room temperature. Blood cells and clotted blood were separated from serum by centrifugation at 1000×*g* for 10 min at 23 °C and serum was stored at − 80 °C.

### Preparation of EV samples from human milk and serum

EVs of paired milk and serum samples were isolated using an identical protocol^16^, with minor modifications. In short, equal volumes of stored cell and fat-free milk supernatant and serum (2.5–3 ml) were thawed at 37 °C, transferred into polyallomer SW60 tubes (Beckman Coulter) and centrifuged at 4 °C for 30 min at 5000×*g* and subsequently at 10,000×*g* (Beckman Coulter Optima XPN-80 with a SW60 rotor). Next, 3.5–4 ml aliquots of the resulting 10,000x*g* supernatants were added up with 3–2.5 ml 1X PBS (Gibco, Carlsbad, CA, USA) to reach a final volume of 6.5 ml and were transferred on top of a 60–10% iodixanol gradient (Progen Biotechnik GmbH, Heidelberg, Germany) in a polyallomer SW40 tube (Beckman Coulter). Gradients were ultracentrifuged at 192,000×*g* for 16–18 h at 4 °C (Beckman Coulter Optima XPN-80 with a SW40Ti rotor). Fifteen gradient fractions of 500 μl were collected and EV-containing fractions (densities 1.06–1.19 g/ml) were pooled^15,16^. Subsequently, the sample was further purified and Iodixanol was removed by size exclusion chromatography using a 20 ml column (Bio-Rad Laboratories, Hercules, CA, USA) packed with 20 ml Sephadex *g* 100 (Sigma-Aldrich, St. Louis, MO, USA). Fractions of 1 ml were eluted with phenol red free RPMI 1640 medium (Gibco, Carlsbad, CA, USA). EV-containing eluates 3–9 (7 ml in total) were pooled^16^, aliquoted and frozen at − 80 °C until further use.

### Nanoparticle Tracking Analysis (NTA)

Purified milk and serum EV preparations were quantified by NTA using a NanoSight NS300 instrument (Malvern Instruments Ltd, UK) equipped with a 405 nm laser and a high sensitivity sCMOS camera. The NanoSight NTA software version 3.2.16 was used for data acquisition and processing. A NanoSight Syringe Pump (Malvern Instruments Ltd, UK) was used for flow mode measurements, which allows a continuous slow flow of the sample through the measurement chamber. Milk EV and serum EV samples required respectively 1:100 and 1:10–1:50 dilution in endotoxin-free Dulbecco’s 1X PBS without Ca^++^ and Mg^++^ (TMS-012-A Millipore) to reach the recommended concentration of 1 × 10^8^–1 × 10^9^ particles/ml (corresponding to particles in frame between 10 and 100). For each EV sample n = 3 60 s video captures were acquired with a syringe pump flow rate of 20 AU and controlled temperature set at 20 °C. Captures were obtained with camera level 13, camera shutter 1232 and camera gain 219 (default). Capture processing was performed with detection threshold 9, viscosity set to water (0.9cP) and automatic blur and minimum track length. The minimum number of valid tracks per EV sample was set at 1000, as recommended^22^.

### Multiplex bead-based flow cytometric analysis of EV surface proteins by MACSPlex Exosome Kit

Purified milk and serum EVs were subjected to multiplex bead-based analysis by flow cytometry using the human MACSPlex Exosome Kit (Miltenyi Biotec, 130-108-813). EV samples were processed overnight in the MACSPlex Filter Plate as described in the manufacturer’s instructions and in a previously published paper on a systematic evaluation of the assay^21^. Particle counts determined by NTA were used to calculate the input amount of EVs. For the optimization experiment five different input numbers of EVs were chosen in the range of 10^8^–10^9^ for milk EVs and 5 × 10^7^–5 × 10^8^ for serum EVs. All subsequent experiments were performed with 5 × 10^8^ EVs as input for both milk and serum EVs. To achieve this, the volume of milk and serum EV samples containing the appropriate number of EVs was diluted with MACSPlex buffer to a final volume of 120 μl and added to pre-wet and drained wells of the MACSPlex 96-well 0.22 μm filter plate. 15 μl of MACSPlex Exosome Capture Beads (containing 39 antibody-coated, hard-dyed bead populations) were added to each sample for overnight incubation (17–18 h) on an orbital shaker at room temperature protected from light. Beads were washed by adding 200 μl of MACSPlex buffer to each sample and wells were drained by centrifuge at 300×*g* for 3 min at 23 °C. For detection of EVs bound to capture beads, 5 μl of APC-conjugated CD9, CD63 and/or CD81 detection antibodies were added to each well containing 135 μl of MACSPlex buffer and incubated for 1 h on an orbital shaker at room temperature protected from light. Antibody clones and concentrations used in the kit are proprietary knowledge of Miltenyi Biotec. EV detection was performed with the three tetraspanin detection antibodies used separately (single detection) or together (pan-tetraspanin detection). Samples were washed with 200 μl of MACSPlex buffer and centrifuged at 300×*g* for 3 min at 23 °C. Next, samples were resuspended in 200 μl of MACSPlex buffer, incubated for 15 min on an orbital shaker at room temperature protected from light and centrifuged at 300×*g* for 3 min at 23 °C. Finally, samples were resuspended in 150 μl of MACSPlex buffer and transferred to a V-bottom 96-well plate for flow cytometric analysis. As negative control RPMI diluted in MACSPlex buffer underwent the same procedure as EV samples to determine non-specific signals. Per well, 135 μl were acquired corresponding to 8000–17,000 recorded beads (Supplementary Table S2). The 39 capture bead populations were distinguished from each others by the 488 nm laser (optical filter 525/40 nm, FITC) and the 561 nm laser (optical filter 585/42 nm, PE), and EVs were detected using the 638 nm laser (optical filter 660/10 nm, APC) of a CytoFLEX LX Flow Cytometer (Beckman Coulter). For data acquisition CytExpert software version 2.1 was used. FlowJo™ software version 10.8.1 (BD Life Sciences) was used for flow cytometry data analysis. Processing of the raw APC-median fluorescence intensity (MFI APC) of all 39 capture bead populations was performed as follows: background correction was performed by subtracting the respective MFI APC values of matched buffer controls, as well as the fluorescent intensity of the corresponding isotype controls (mIgG1, REA). Protein signals with MFI > MFI of isotype controls were considered positive. The other proteins were not used for further analyses. For the generation of part-of-a-whole graphs, to assess the cell origin of EVs, background and isotype subtracted MFI were normalized to 100.

### Statistical analysis

All statistical analyses were performed with GraphPadPrism 9.0.0 (GraphPad Software, San Diego, California USA). Data normality was assessed by Shapiro–Wilk test and d’Agostino-Pearson test. Non-normally distributed variables were expressed as median and analysed by Mann–Whitney test or Kruskal–Wallis test followed by Dunn’s multiple comparison, as appropriate. Paired samples were analysed by Wilcoxon test with FDR. A probability value of < 0.05 was considered statistically significant.

## Discussion

In this study we used the multiplex bead-based flow cytometry MACSPlex assay to characterize the surface protein profiles of purified EVs, isolated with the same protocol from paired human milk and serum samples. Since 2018, the MACSPlex assay has been used for surface protein profiling of EVs from various body fluids, i.e. blood plasma^21,37–41^, serum^21,42–44^, urine^44^, cerebral spinal fluid^38^ and lymphatic drain fluid^45^ of healthy subjects and patients, using a wide variety of EV enrichment/purification protocols, EV quantification methods and input EV amounts. To our knowledge no studies have been reported on the use of this assay on EVs purified from different body fluids collected on the same day from the same donor, isolated by similar protocols and analysed by pan-tetraspanin (simultaneous detection of CD9, CD63 and CD81) as well as single tetraspanin detection. Analysis by pan-tetraspanin detection of EVs bound to CD9, CD63 and CD81 capture beads showed comparable detection signals for milk EVs and serum EVs bound to CD9 and CD63 beads, while the signal for CD81 capture beads was significantly higher in milk EVs compared to serum EVs. By using the same number of input EVs, the higher CD81 signal on milk EVs could reflect either a higher number of CD81^+^ EVs in milk compared to serum, more CD81 molecules per EV or the presence of EV subsets with high expression levels of CD81, which can only be deciphered by performing single EV-based analysis Previously, it has been reported that CD81 was not present on 200,000×*g*/100 nm EVs isolated from different cell lines, while it was detectable on 100,000×*g*/200 nm EVs, suggesting that CD81 was expressed on larger particles^46^. Accordingly, NTA-based size distribution of EVs used in our study show that milk EVs (234 ± 13 nm) are larger than serum EVs (105 ± 10 nm). Moreover, it was shown by flow cytometry that CD81 MFI signal was 100–1000 times higher in milk EVs compared to EVs purified from plasma^47^, and that CD81^+^ EVs were the rarest EV population in plasma and serum^48^. Furthermore, it has been reported that platelets do not express CD81^49^ and accordingly, platelet-derived EVs were hardly detected with CD81 beads^23^ and were negative in CD81 by westernblotting^50^. As expected, our data show an enrichment of proteins associated with platelets and platelets activation in serum EVs, such as CD41b, CD42a, P-selectin/CD62P and PECAM-1/CD31^50^, an indicator for the presence of platelets-derived EVs. Besides the presence of platelet-derived EVs in serum, we also identified EVs derived from immune cells, albeit their contribution to the total detection signal in the assay was much lower. Also in milk EVs we could detect clear signatures of immune cells-derived EVs, but not blood signatures, thus suggesting that EVs from milk are not likely to come directly from blood.

In line with previous research^51,52^, our data show that milk EVs carry HLA-II, CD14, CD24 and CD3 which reinforce the hypothesis of transfer of immunological features by EVs from mother to infant. In accordance with our previous data^52^, besides immune cells-derived EVs we could detect protein signatures that could be linked to breast luminal epithelial cells-derived EVs or mesenchymal milk stem cells-derived EVs in milk. Of note, most proteins are not exclusively expressed on one cell type, i.e. CD14 is described as marker of monocytes but is also expressed on mammary epithelial cells^31^. Similarly, CD24 has been associated to both B cells and luminal epithelial cells^32^, and both CD24 and CD326 (EpCAM) are highly expressed on lactocytes^8,28^. Our analysis of milk EVs showed that CD24 and CD326 were two of the highest expressed EV proteins. For defining the potential cells of origin of EVs in milk and serum, we selected the most likely cellular source. However, we cannot exclude that some EVs, expressing markers present on a variety of different cell types, can be derived from other cell types than the ones indicated in our hypothetical model.

Since in our study we analysed milk and serum samples derived from non-allergic and allergic donors, we also evaluated whether the surface protein profiles of milk and serum EVs identified in our study differed between non-allergic (n = 5) and allergic (n = 4) donors. Although, we observed a tendency that the detection of EVs captured by some protein markers on allergic milk EVs was lower than for non-allergic milk EVs, while this was not observed for serum EVs expressing the same marker, these differences were not significant. However, since we tested only a small number of donors we cannot exclude that upon testing more donors significant differences between milk EVs from non-allergic and allergic donors can be unveiled. Be that as it may, despite the use of relatively small donor numbers, we found clear significant differences between milk and serum-derived EVs, demonstrating the robustness of the identified body fluid specific-EV protein signatures.

As demonstrated in our current study, besides pan-tetraspanin EV detection, single tetraspanin-detection in relation to specific protein markers can yield important insights on EVs, however this approach is frequently disregarded. By comparing pan-tetraspanin detection and single tetraspanin detection, here we unveiled characteristic tetraspanin profiles for different protein capture bead populations. For example, serum EVs captured with the antigen-presenting cell marker HLA-II expressed all three tetraspanins CD9, CD63 and CD81, which might suggest that HLA-II^+^ EVs originate and are released via the endosomal route. Conversely, serum EVs expressing the antigen-presenting cell associated co-stimulatory protein CD40 were exclusively detected with αCD9 antibody, hinting at their origin from the plasma membrane. These findings suggest the presence of different subsets of antigen-presenting cells-derived EVs in serum and milk. On the other side, T cell-associated markers CD3 detected on milk EVs, and CD8 detected on serum EVs were characterized by the same tetraspanin distribution, i.e. positive for CD81 and CD63 but negative for CD9, suggesting that these T cells-derived EVs are both from endosomal origin. Finally, milk EVs captured by HLA-I did not express CD63, as opposite to a previous study^53^ showing that CD63-bead bound milk EVs and HLA-I-bead bound milk EVs positively stained for HLA-I and CD63, respectively. This discrepancy can be caused by differences in milk biobanking protocols, e.g. biobanking full milk^53^ versus cell and fat-depleted milk^18^, EV sample preparation, or affinity/avidity for the antibodies used.

In conclusion, by using the MACSPlex protein profiling assay in conjunction with both pan-tetraspanin and single tetraspanin detection we were able to define body fluid-specific protein signatures on EVs and detailed tetraspanin-signatures for EV subsets expressing a certain surface protein. The detailed information on specific tetraspanin distribution in conjunction with surface proteins of interest can be exploited for the identification of combined protein-biomarkers. Furthermore, designing tailored detection strategies, e.g. composing antibody detection cocktails for selected tetraspanins and other membrane proteins of interest, will further refine the definition of combined protein-biomarkers for more robust EV subsets of interest. Moreover, the insights on body fluid-specific protein signatures can be used to exclude EV subsets in body fluids that can potentially mask EVs of interest, thus greatly facilitating the analysis of rare EV subsets according to the principle “remove the hay to find the needle”.

## Supplementary Information

Supplementary Information.

## Data Availability

Raw multiplex data are shared in Supplementary Tables. Additional information for reanalysis may be requested from the lead contact. We have submitted all relevant data of our experiments to the EV-TRACK knowledgebase^54^ (EV-TRACK ID: EV220315).
