# Supplementary material for: Surface protein profiling of milk and serum extracellular vesicles unveils body fluid-specific signatures
Source: Sci Rep. 2023 May 30;13:8758. doi: 10.1038/s41598-023-35799-w (PMC10229631; doi:10.1038/s41598-023-35799-w)
Supplement: Supplementary file 1 — Supplementary Information. [file 41598_2023_35799_MOESM1_ESM.pdf]

Surface protein profiling of milk and serum extracellular vesicles unveils body fluid-specific signatures

Alberta Giovanazzi[1,3], Martijn J.C. van Herwijnen[1], Marije Kleinjan[1], Gerbrich N. van der Meulen[2], Marca H.M. Wauben[1,3*]

1 Department of Biomolecular Health Sciences, Faculty of Veterinary Medicine, Utrecht University, Utrecht, The Netherlands

2 Paediatric Allergy Centre, Department of Paediatrics, Martini Hospital, Groningen, The Netherlands

3 TRAIN-EV Marie Skłodowska-Curie Action-ITN

***** m.h.m.wauben@uu.nl

SUPPLEMENTARY INFORMATION

Supplementary Table S1. Clinical parameters of milk and serum donors									
Donor number	***1***	***2***	***3***	***4***	***5***	***6***	***7***	***8***	***9***
Moternal age *(years)*	30	34	34	31	32	36	25	31	33
Lactational stage *(weeks post-partum)*	9	5	6	5	6	8	7	5	6
Number of full term deliveries	2	4	2	2	2	1	1	1	3
Health status	A	NA	A	NA	A	NA	A	NA	NA
Total IgE *(kU/L)*	73.6	24.7	243	27.8	141	38.9	81.9	30.4	9.6
grass pollen IgE *(kU/L)*	< LOD	< LOD	0.97	< LOD	< LOD	< LOD	1.3	< LOD	< LOD
tree pollen IgE *(kU/L)*	7.6	< LOD	40	< LOD	< LOD	< LOD	7.7	< LOD	< LOD
house dust mite IgE *(kU/L)*	1.0	< LOD	4.2	< LOD	3.8	< LOD	4.4	< LOD	< LOD
cat dander IgE *(kU/L)*	< LOD	< LOD	< LOD	< LOD	< LOD	< LOD	0.49	< LOD	< LOD
dog dander IgE *(kU/L)*	< LOD	< LOD	< LOD	< LOD	< LOD	< LOD	1.1	< LOD	< LOD

LOD= limit of detection
A= allergic
NA=non-allergic

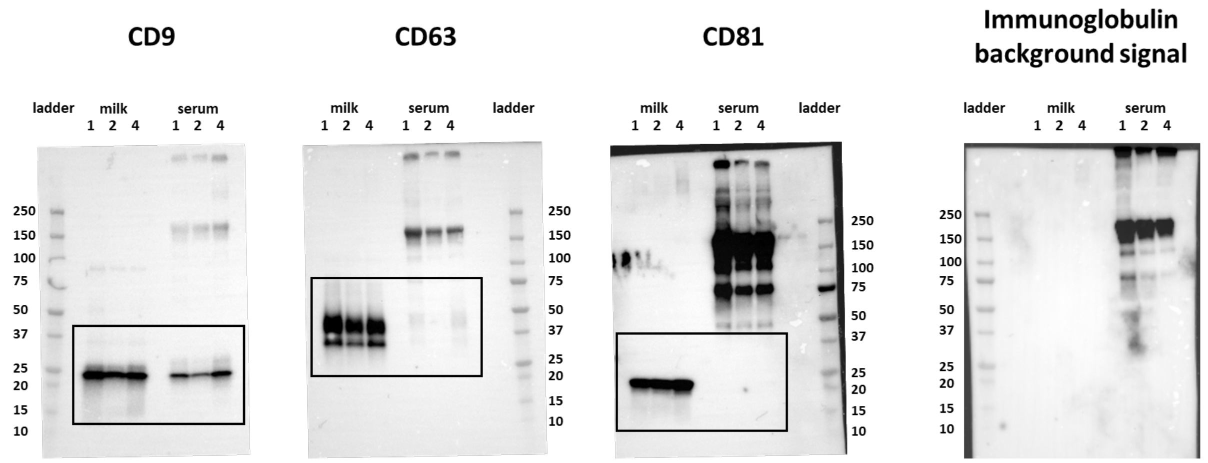

Supplementary Figure S1. Quality controls of EV samples by Western blotting for tetraspanins. Isolated EV samples from 3 different paired milk and serum donors (see Supplementary Table S1 for information on Donor 1, 2 and 4) were analysed by Western blotting (non-reduced conditions) for the presence of tetraspanin CD9 (24 kDa, exposure time 12 s), CD63 (37 kDa, exposure time 120 s), CD81 (26 kDa, exposure time 300 s) and immunoglobulin background signals (No primary antibody, only secondary goat-anti-mouse IgG-HRP. Heavy H chain: 50 kDa, light L chain: 25 kDa, 2H+2L= IgG: 150 kDa, 2H= 100 kDa, H+L= 75 kDa, exposure time 15 s). Input material of donors 1, 2 and 4 was normalised based on particle numbers as measured by NTA (Fig 1a) and $11.5*10^9$ particles were used of each sample for Western blot analysis. Hereto, corresponding volumes of isolated EV samples were pelleted for 35 min at 100,000x*g* (in a Beckman Coulter Optima Max-XP with a TLA-55 rotor) in polyallomer microcentrifuge tubes (Beckman). The pellet was resuspended in sample buffer (62.5 mM Tris pH 6.8, 2% SDS, 10% Glycerol), heated at 95°C for 3 min, and run on an 8%–16% TGX-Criterion gel (Bio-Rad). The separated proteins were transferred to PVDF membranes and blocked in PBS containing 0.2% fish skin gelatin (Sigma-Aldrich) and 0.1% Tween-20. Proteins were detected by immunoblotting using mouse anti-human CD9 (clone HI9a, BioLegend, dilution 1:1,000), mouse anti-human CD63 (clone TS63, Abcam, dilution 1:1,000), or mouse anti-human CD81 (clone JS64, Immunotec, dilution 1:1,000). Goat anti-mouse-HRP (Jackson Immuno Research, Suffolk, UK; dilution 1:10,000), was used as secondary antibody. HRP conjugated antibodies were detected using SuperSignal West Dura Chemiluminescent Substrate (Thermo Scientific and ChemiDoc XRS and Image Lab 5.1 (Bio-Rad).

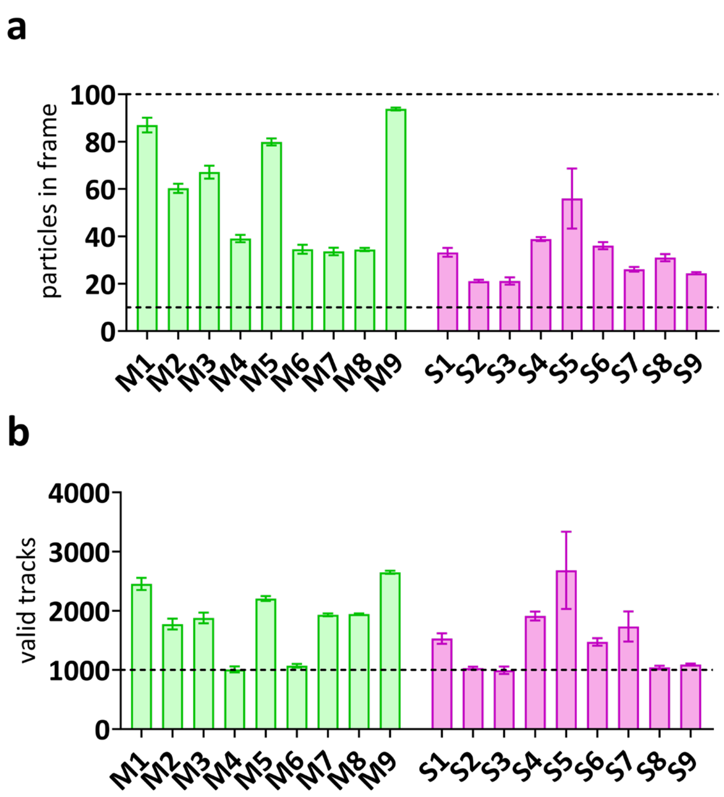

Supplementary Figure S2. Technical quality controls for NTA. To ensure reliable measurements, milk and serum EVs were diluted to reach 10-100 particles in frame **(a)** and at least 1,000 valid tracks as average of 3 captures per EV sample (**b**). The results shown are derived from n=9 individual milk samples (M1-M9) and paired serum samples (S1-S9).

Supplementary Table S2. Raw bead counts and percentages of capture signals

samples	acquired events (counts)	beads (counts)	single beads (counts)	APC+ beads (FITC) (counts)	APC+ beads (PE) (counts)	APC+ beads (FITC) (% of single beads)	APC+ beads (PE) (% of single beads)
buffer	17256	15947	15929	196	195	1.23	1.22
10^9 milk 1	15579	11302	11077	3415	3412	30.8	30.8
10^9 milk 2	17325	13074	13007	4074	4065	31.3	31.3
10^9 milk 3	18106	13349	13290	4527	4522	34.1	34
7.5*10^8 milk 1	16533	12543	12415	3344	3333	26.9	26.8
7.5*10^8 milk 2	18137	14292	14240	4129	4118	29	28.9
7.5*10^8 milk 3	17686	13392	13273	4198	4189	31.6	31.6
5*10^8 milk 1	19146	14980	14805	3508	3494	23.7	23.6
5*10^8 milk 2	17308	13267	13227	3359	3351	25.4	25.3
5*10^8 milk 3	21644	17311	17156	5055	5049	29.5	29.4
2.5*10^8 milk 1	12010	8988	8884	1925	1926	21.7	21.7
2.5*10^8 milk 2	11750	8779	8764	2040	2037	23.3	23.2
2.5*10^8 milk 3	10885	7913	7790	2119	2117	27.2	27.2
10^8 milk 1	14984	11252	11196	2286	2278	20.4	20.3
10^8 milk 2	16818	12852	12792	2629	2621	20.6	20.5
10^8 milk 3	16757	13063	13010	2955	2948	22.7	22.7
5*10^8 serum 1	18031	12344	12000	3784	3766	31.5	31.4
5*10^8 serum 2	14758	10369	10316	4004	3992	38.8	38.7
5*10^8 serum 3	19132	13693	13530	4035	4023	29.8	29.7
2.5*10^8 serum 1	17481	12305	12041	2856	2845	23.7	23.6
2.5*10^8 serum 2	15387	11029	10974	3400	3379	31	30.8
2.5*10^8 serum 3	16943	12510	12120	2928	2925	24.2	24.1
10^8 serum 1	17796	13336	13230	2291	2280	17.3	17.2
10^8 serum 2	15949	11959	11711	2584	2569	22.1	21.9
10^8 serum 3	13313	9847	9512	1972	1966	20.7	20.7
7.5*10^7 serum 1	15056	10801	10566	1566	1557	14.8	14.7
7.5*10^7 serum 2	13441	9759	9590	1859	1846	19.4	19.2
7.5*10^7 serum 3	11509	8257	8053	1552	1549	19.3	19.2
5*10^7 serum 1	18373	13975	13843	1722	1710	12.4	12.4
5*10^7 serum 2	18019	14171	14001	1638	1618	11.7	11.6
5*10^7 serum 3	15502	12136	11801	1875	1868	15.9	15.8

Supplementary Table S2. Raw bead counts and percentages of MACSPlex bead capture signals. Acquired events is the number of events recorded in 135 µl of volume at medium flow rate (30 sec/min). Beads were gated and single beads were the events used as input for the analysis of count or relative percentage of APC^+ bead populations (EVs bound to beads). Beads are distinguished from each others by their respective fluorescence characteristics using the lasers and optical filters for FITC and PE. Data were obtained from the gating strategy in Supplementary Fig. S2a. Data are shown in the bar graphs of Supplementary Fig. S2b.

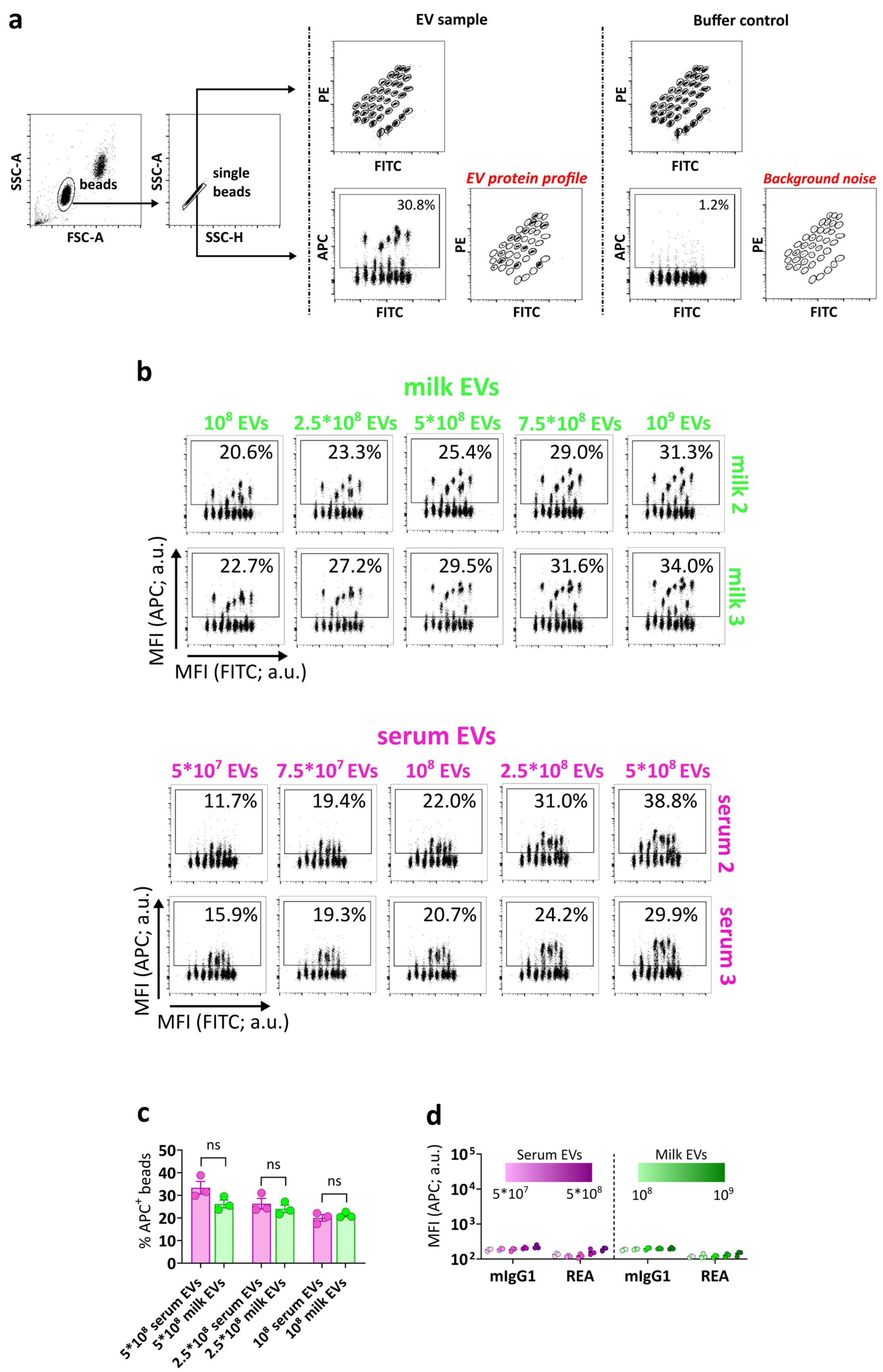

Supplementary Figure S3. Titration of APC detection signals is proportional to input EV numbers. (a) Overview of the MACSPlex gating strategies: events in 135 ul were recorded at medium flow rate

(30sec/min). Beads were gated and single beads were used as input for the analysis of APC^+ bead populations (i.e. EVs bound to beads and detected by APC-conjugated tetraspanin antibodies). Beads were distinguished from each others based on their specific FITC and PE signals. **(b)** Flow cytometry dot plots of five input amounts of milk EVs (green) and serum EVs (pink) from donor 2 and 3 depicting the percentage of APC^+ beads (relative to single beads). See Supplementary Table S2 for further details on raw bead counts and percentage of capture signals. **(c)** Technical control: percentage of APC^+ beads (EVs bound to beads) in relation to input number of milk EVs and serum EVs. The graph depicts median with dots of donor 1, donor 2, donor 3. **(d)** Technical control: MFI APC relative to internal isotype controls mIgG1 and REA capture beads at different EV doses. The graph depicts median + min and max values (n=3 donors). a.u. = arbitrary unit.

Supplementary Table S3: Raw MFI APC signal (pan-tetraspanin) of all 39 beads

	buffer	*M1*	*M2*	*M3*	*M4*	*M5*	*M6*	*M7*	*M8*	*M9*	*S1*	*S2*	*S3*	*S4*	*S5*	*S6*	*S7*	*S8*	*S9*
CD1c	125	125	195	291	213	305	195	245	172	220	222	1640	271	959	538	228	327	233	322
CD2	141	204	258	428	297	193	485	314	310	243	202	267	182	209	239	254	265	226	228
CD3	432	820	1918	1804	2089	2403	680	2023	1055	1399	295	479	316	228	314	258	248	191	274
CD4	125	125	171	241	169	116	222	172	230	151	176	152	149	149	189	176	169	165	180
CD8	101	204	308	422	250	211	444	282	261	252	1432	2314	1491	1333	1281	1766	1097	1618	1523
CD9	125	6762	6639	14726	7534	10658	26482	8171	33644	8019	5754	7724	13842	2740	9595	6762	14871	3230	14969
CD11c	138	149	132	250	149	187	151	178	176	141	853	263	231	140	176	140	169	165	140
CD14	305	24353	25099	54009	37119	33305	38141	32746	126955	15878	485	639	687	511	698	399	538	553	493
CD19	143	163	200	217	217	333	198	196	178	136	280	513	274	259	728	198	261	352	263
CD20	280	343	373	493	446	570	446	434	483	348	350	731	375	348	738	327	389	432	316
CD24	162	16735	6659	41808	11510	29196	26042	17013	40547	14207	1012	970	522	905	3167	790	978	950	1257
CD25	401	195	219	356	346	547	219	288	254	196	273	651	263	348	858	195	274	341	224
CD29	116	967	918	3028	1287	2651	6245	1020	4442	1507	2786	6700	6439	1091	7772	4058	6170	1115	4390
CD31	172	172	198	387	195	329	436	436	442	176	1052	761	1693	538	4574	1257	1819	479	2175
CD40	256	267	284	493	274	339	717	335	405	288	937	992	2163	409	4301	850	1322	507	2615
CD41b	337	373	362	517	360	678	474	440	553	360	8755	6226	6558	2163	14823	12356	15878	3690	19741
CD42a	118	156	101	147	140	198	105	132	151	138	8978	5806	23008	1766	23790	12080	26042	2320	27204
CD44	258	373	339	557	401	466	1106	448	604	358	393	578	540	483	2538	735	1094	528	761
CD45	269	282	293	483	312	352	430	368	430	288	411	362	483	401	1644	551	604	345	931
CD49e	215	193	184	265	213	182	200	213	196	228	261	211	219	217	750	207	335	200	356
CD56	671	815	626	664	566	800	624	825	666	559	428	507	454	432	364	393	354	322	430
CD62P	97.7	202	81.4	95.9	81.4	134	85	92.3	120	86.8	7126	5949	17525	964	15723	11111	24682	3765	21667
CD63	230	6867	6931	22179	9504	11147	11510	6095	36619	7039	10225	17525	25954	3341	19417	10899	13797	6598	22105
CD69	184	299	289	576	545	1026	312	379	639	239	479	1523	553	327	1287	325	540	572	481
CD81	191	15569	20681	33758	22253	25695	55509	21098	64997	19035	9595	8069	9223	6303	10624	8171	9841	9092	10726
CD86	209	265	312	474	379	624	341	329	343	245	243	524	271	269	460	243	295	671	245
CD105	196	452	356	810	515	778	622	430	2371	368	1067	669	669	553	717	485	628	454	530
CD133/1	239	30197	10323	43701	13355	12119	29000	5516	180227	17994	387	1184	680	515	918	549	648	581	476
CD142	274	346	337	454	373	389	442	391	495	325	265	314	360	291	324	293	295	335	284
CD146	235	918	983	1519	771	1593	3659	1165	1954	931	609	440	515	393	381	391	327	375	345
CD209	94.1	94.1	138	154	101	123	118	136	132	120	112	187	114	95.9	158	77.8	95.9	174	120
CD326	585	49245	62803	86494	44452	65897	161834	54195	109019	56857	511	3909	921	622	781	491	517	873	505
mIgG1	202	191	222	370	220	306	362	377	358	217	191	256	200	312	413	295	343	333	202
REA	132	149	147	158	136	180	101	149	180	143	182	226	189	125	191	118	123	136	143
HLA-I	462	850	738	1539	611	581	4494	637	1109	536	992	902	1134	589	2961	1555	3456	553	2470
HLA-II	171	16516	21885	54942	30710	9873	127396	12003	87999	16035	1653	3114	2637	3062	2234	2175	3322	2192	3131
MCSP	202	206	248	297	276	385	278	267	252	217	233	520	289	250	377	246	278	346	256
ROR1	213	915	1503	4058	1893	2817	16194	1200	3408	2371	1274	3071	745	820	1734	405	764	1124	1152
SSEA-4	368	424	397	460	458	752	356	297	470	413	301	764	411	284	530	258	288	310	446

Supplementary Table S3. Raw MFI APC signal (pan-tetraspanin) of all 39 bead populations of the MACSPlex Exosome kit. Raw MFI APC intensities of each capture bead population without background and isotype corrections are indicated. M1-M9 refers to milk EV samples of n=9 individual donors, while S1-S9 are the paired serum EV samples.

Supplementary Table S4_Dunn's multiple comparisons test isotype VS proteins

milk

	Mean rank diff.	Adj p value	Significant
CD1c	-62	>0.99	ns
CD2	-107	0.91	ns
**CD3**	**-198**	**0.001**	**
CD4	-36	>0.99	ns
CD8	-126	0.29	ns
**CD9**	**-246**	**<0.001**	***
CD11c	-9.9	>0.99	ns
**CD14**	**-276**	**<0.001**	***
CD19	-50	>0.99	ns
CD20	-107	0.88	ns
**CD24**	**-260**	**<0.001**	***
CD25	-12	>0.99	ns
**CD29**	**-213**	**<0.001**	***
CD31	-93	>0.99	ns
CD40	-59	>0.99	ns
CD41b	-124	0.32	ns
CD42a	-51	>0.99	ns
CD44	-144	0.09	ns
CD45	-56	>0.99	ns
CD49e	0	>0.99	ns
CD56	-50	>0.99	ns
CD62P	-25	>0.99	ns
**CD63**	**-245**	**<0.001**	***
CD69	-135	0.17	ns
**CD81**	**-269**	**<0.001**	***
CD86	-92	>0.99	ns
**CD105**	**-174**	**0.009**	**
**CD133-1**	**-261**	**<0.001**	***
CD142	-88	>0.99	ns
**CD146**	**-203**	**<0.001**	***
CD209	-25	>0.99	ns
**CD326**	**-291**	**<0.001**	***
**HLA-I**	**-172**	**0.01**	*
**HLA-II**	**-269**	**<0.001**	***
MCSP	-46	>0.99	ns
**ROR1**	**-218**	**<0.001**	***
SSEA-4	-101	>0.99	ns

serum

	Mean rank diff.	Adj p value	Significant
CD1c	-113	0.62	ns
CD2	-45	>0.99	ns
CD3	0	>0.99	ns
CD4	-26	>0.99	ns
**CD8**	**-210**	**<0.001**	***
**CD9**	**-264**	**<0.001**	***
CD11c	-47	>0.99	ns
CD14	-115	0.58	ns
CD19	-88	>0.99	ns
CD20	-69	>0.99	ns
**CD24**	**-189**	**0.002**	**
CD25	-29	>0.99	ns
**CD29**	**-240**	**<0.001**	***
**CD31**	**-194**	**0.002**	**
**CD40**	**-180**	**0.005**	**
**CD41b**	**-265**	**<0.001**	***
**CD42a**	**-271**	**<0.001**	***
**CD44**	**-152**	**<0.05**	*
CD45	-114	0.6	ns
CD49e	-45	>0.99	ns
CD56	0	>0.99	ns
**CD62P**	**-267**	**<0.001**	***
**CD63**	**-278**	**<0.001**	***
CD69	-143	0.09	ns
**CD81**	**-270**	**<0.001**	***
CD86	-67	>0.99	ns
**CD105**	**-152**	**<0.05**	*
**CD133-1**	**-147**	**<0.05**	*
CD142	-23	>0.99	ns
CD146	-83	>0.99	ns
CD209	-29	>0.99	ns
CD326	-58	>0.99	ns
**HLA-I**	**-179**	**0.006**	**
**HLA-II**	**-231**	**<0.001**	***
MCSP	-50	>0.99	ns
**ROR1**	**-184**	**0.004**	**
SSEA-4	-38	>0.99	ns

Supplementary Table S4. Statistical analysis to define proteins below the isotype threshold (non-detected) and above the isotype threshold (expressed on EVs). Significance was determined by non-parametric Kruskal-Wallis Test with post-hoc Dunn's multiple comparison test isotype VS protein signals.. Significance: ns (p≥0.05), * (p<0.05), ** (p<0.01), *** (p<0.001).

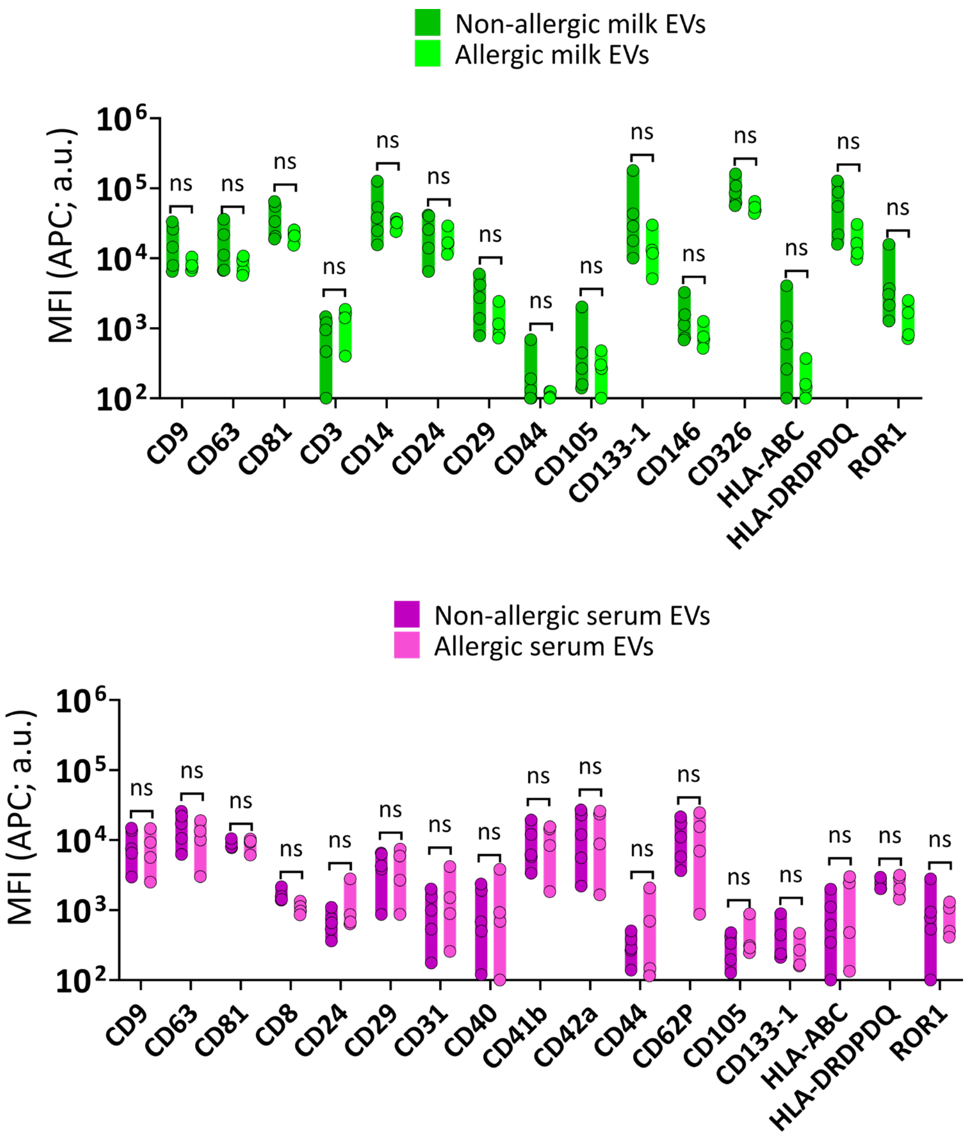

Supplementary Figure S4. Comparison of EVs from non-allergic and allergic donors. MFI APC signals of pan-tetraspanin detection associated to surface proteins identified in milk and/or serum EVs (Figure 3) was compared between milk EVs and serum EVs from non-allergic (n=5 donors) and allergic (n=4 donors) mothers. Significance differences between non-allergic and allergic groups was assessed by multiple non-parametric Mann Whitney test with Holm-Šídák multiple comparison method. a.u. = arbitrary unit.

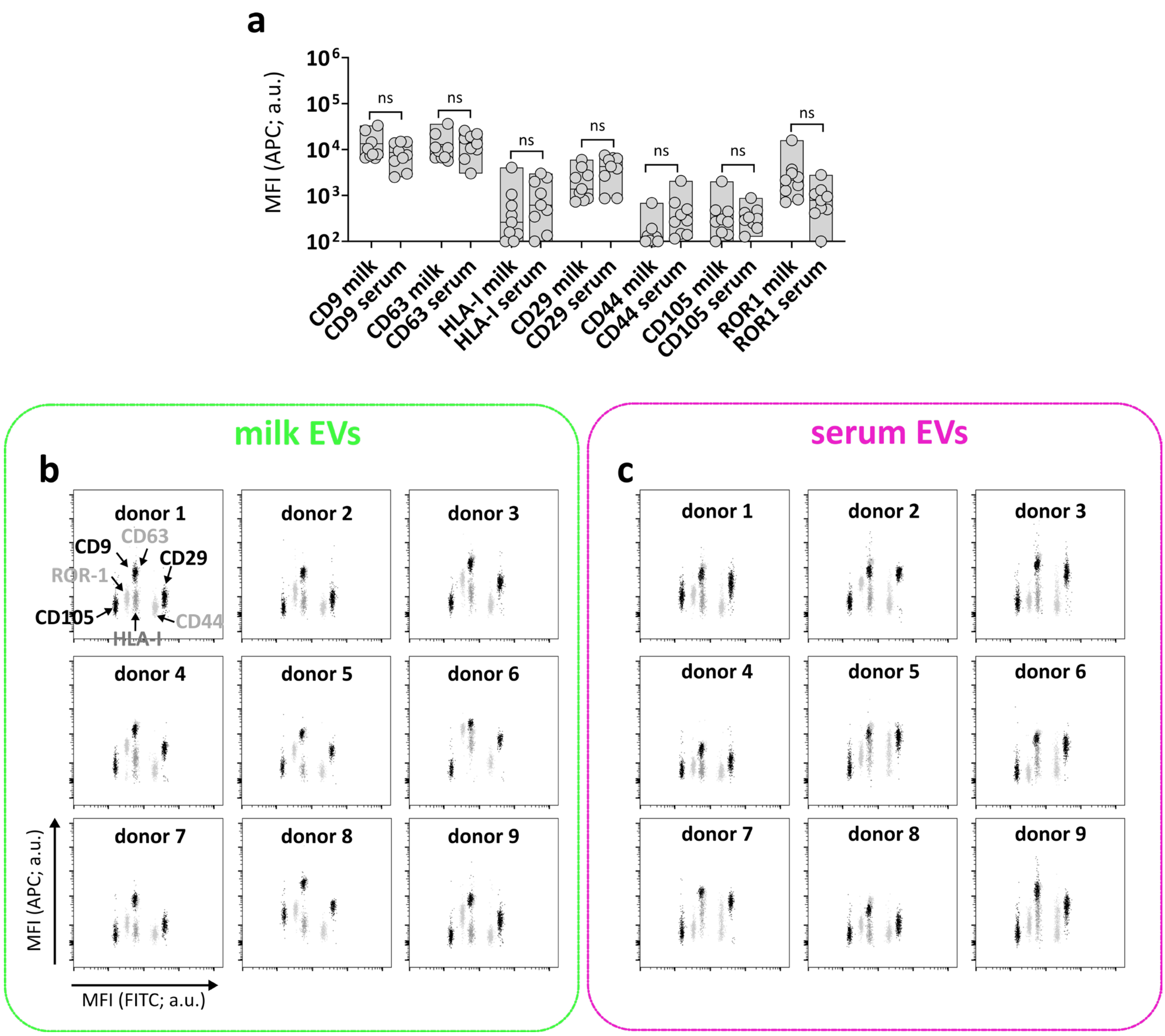

Supplementary Figure S5. Identification of common milk and serum EV proteins. (a) Indicated are the MFI APC pan-tetraspanin signals of n=9 individual EV samples associated to EV proteins similarly expressed on milk and serum EVs. **(b)** Corresponding flow cytometry dotplots of the n=9 individual EV samples of proteins depicted in (a). $p \geq 0.05$ (ns), non-parametric paired T test (Wilcoxon test). a.u. = arbitrary unit.

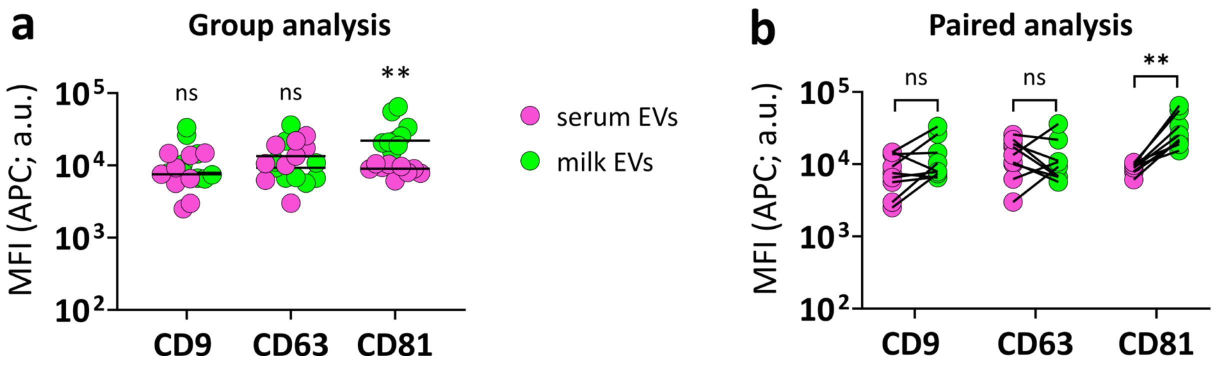

Supplementary Figure S6. Comparison of CD9, CD63 and CD81 bead capture signals in milk and serum EVs with pan-tetraspanin detection. (a) Comparison between milk and serum EVs (n=9 samples/group). Lines represent the medians. **(b)** Paired analysis of milk and serum EVs of individual donors (n=9 donors). $p \geq 0.05$ (ns), $p < 0.01$ (**), Paired T test nonparametric (Wilcoxon test). a.u. = arbitrary unit.

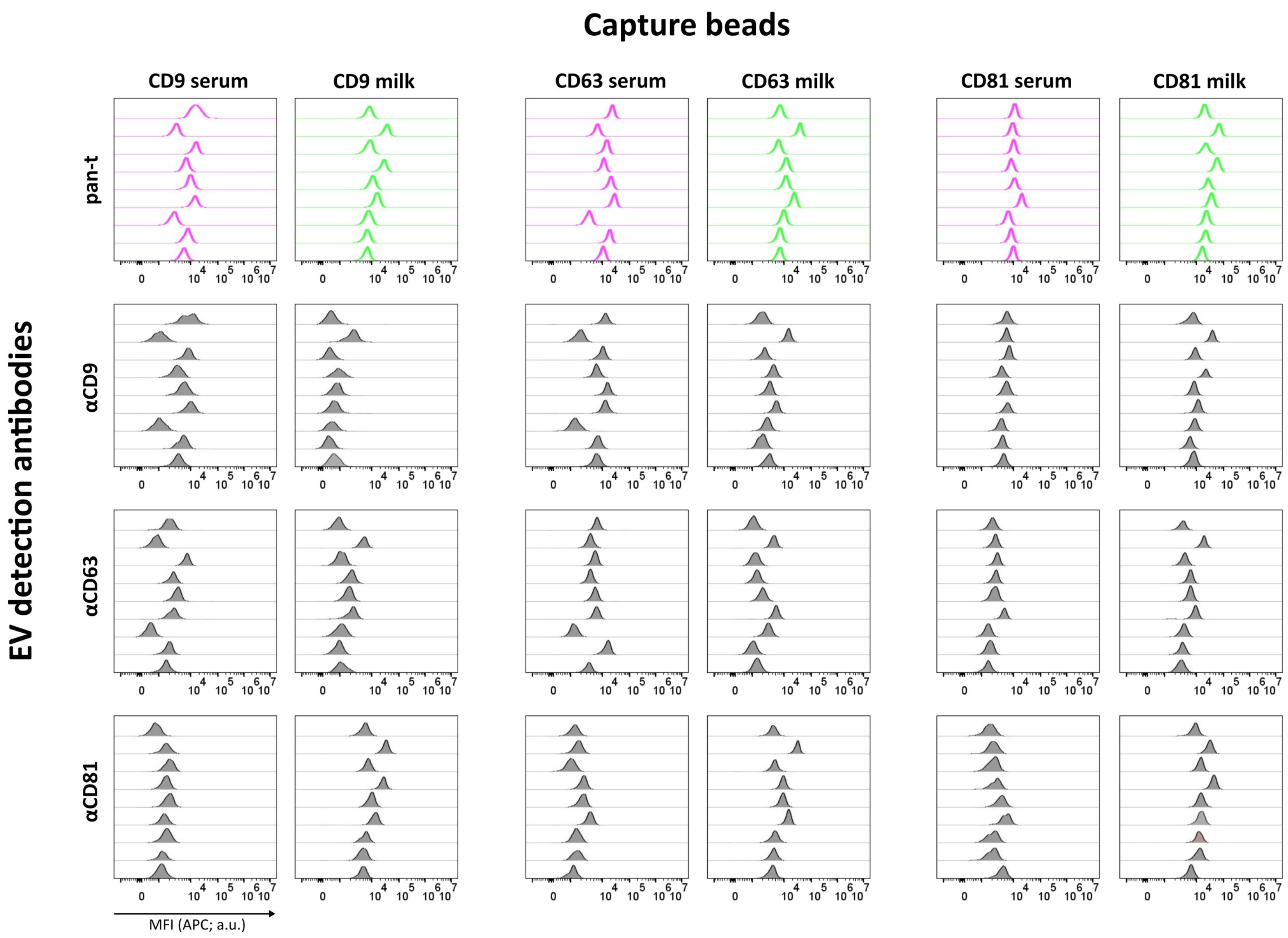

Supplementary Figure S7. Flow cytometry histograms representing MFI APC (x-axis) of CD9, CD63, CD81 capture bead populations incubated with milk EVs or serum EVs (n= 9 donors) and detected with pan-tetraspanin detection (pan-t) or single tetraspanin detection (αCD9, αCD63 or αCD81). a.u. = arbitrary unit.

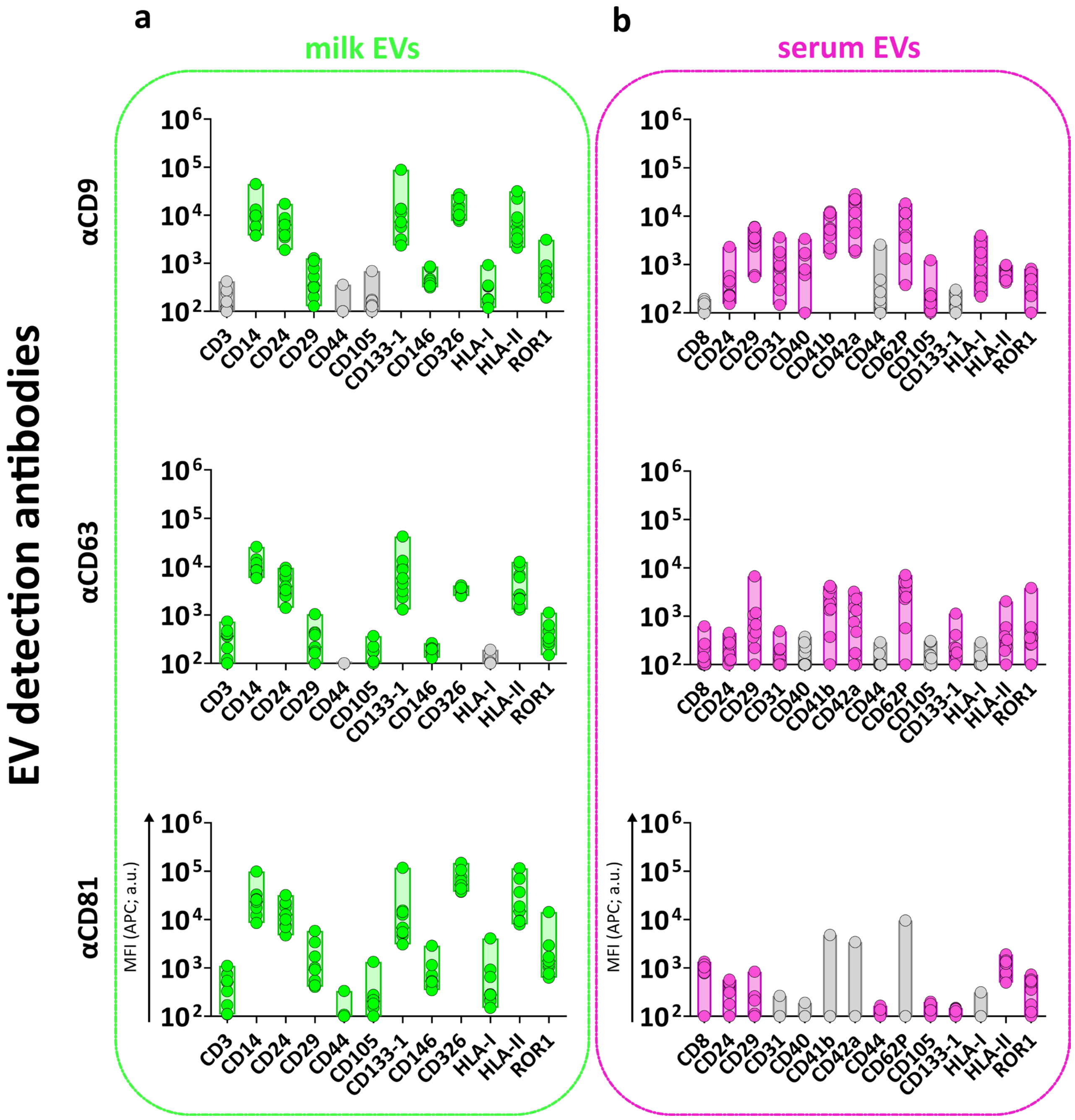

Supplementary Figure S8. The MFI APC of single tetraspanin detection on EVs bound to capture bead populations identified by pan-tetraspanin detection. Single tetraspanin detection was performed with αCD9, αCD63 or αCD81 antibody on milk EVs **(a)** and serum EVs **(b)**. Tetraspanin capture beads CD9, CD63 and CD81 are not included in this figure (Data are presented in Figure 5 and Supplementary Figure S7). Grey bars indicate non-significant detection compared to isotype control. a.u. = arbitrary unit.
